# Landscape associations and population genetics of a generalist carnivore at a range limit

**DOI:** 10.1371/journal.pone.0334492

**Published:** 2025-12-18

**Authors:** Bailey A. Kleeberg, Robert C. Lonsinger, Jennifer R. Adams, Lisette P. Waits, W. Sue Fairbanks

**Affiliations:** 1 Department of Natural Resource Ecology Management, Oklahoma State University, Stillwater, Oklahoma, United States of America; 2 U.S. Geological Survey, Oklahoma Cooperative Fish and Wildlife Research Unit, Oklahoma State University, Stillwater, Oklahoma, United States of America; 3 Department of Fish and Wildlife Sciences, University of Idaho, Moscow, Idaho, United States of America; Wildlife Conservation Society Canada, CANADA

## Abstract

American black bear (*Ursus americanus*) sightings have increased in the Oklahoma Panhandle, an area outside of the species’ historical range, prompting an assessment of bears in the region. We used camera traps and an occupancy modeling framework to identify factors influencing bear detection and space-use patterns. We used noninvasive genetic sampling techniques to evaluate genetic diversity, population structure, and bear abundance in the region. During the summers of 2022–2023, we deployed cameras at 160 sites across western Oklahoma (USA) and detected ≥1 bear at 20 sites. The most-supported model from our single-season single-species analysis indicated that bear detection was positively associated with temperature and precipitation, negatively associated with day of year, and differed between years. The most-supported model indicated that bear space use was negatively associated with elevation (β = −0.013, 85% CI = −0.025, 0.000), and positively associated with slope (β = 0.645, 85% CI = 0.305, 0.984) and coarse woody debris counts (β = 1.539, 85% CI = 0.314, 2.765). We deployed 41 hair snares in Oklahoma resulting in the collection of 153 hair samples and received 69 tissue samples from black bears harvested in northeastern New Mexico. Using 11 microsatellite markers, we identified 21 (12M:9F) bears in western Oklahoma, and 69 (40M:29F) in New Mexico. We found evidence that bears occurring in Oklahoma were an extension of a previously documented population that occurred in northcentral New Mexico. We detected significant population-level heterozygote deficiency (*P* = 0.013) compared to expectations under Hardy-Weinberg equilibrium. Using capture with replacement models, we estimated 26 (95% CI = 19–43) bears in western Oklahoma during 2022–2023. Our results provide baseline data on population distribution, abundance, and genetic health of bears in the region and identify factors that may drive human-bear conflicts as the bear population increases in western Oklahoma.

## Introduction

Environmental change may result in distributional shifts of species to areas where they did not previously occur [1]. Although elevational and latitudinal shifts in species distributions have commonly been associated with climate change [2], distributional expansions or shifts may be facilitated by landscape changes (e.g., land-use change, woody encroachment) that alter the availability of resources [3–5]. Shifts in species distributions tend to occur independently due to species-specific variation in habitat requirements [6]. Still, vagile species with generalist tendencies are predicted to have greater capacity to expand their distribution in response to environmental changes than specialists with lower dispersal capacities [7–9] and environmental change tends to promote generalist species [10]. Contemporary distributional shifts of large carnivores have been predominantly characterized by range contractions [11], but some large carnivores have expanded into portions of their historical range from which they were extirpated [12] or into previously unoccupied areas [13]. Where large carnivores have expanded in distribution, expansions have been attributed to landscape changes including land-cover modifications (e.g., forest regeneration) [14], anthropogenic subsidies [15], or decreased persecution by humans [16]; these changes have often promoted demographic release [17] or occurred in concert with reintroduction efforts [18]. Range expansions of large carnivores have social and political implications, as their presence can elicit an increase in human, economic, or recreational conflicts [19].

Establishing reliable baseline estimates of expanding populations (e.g., space use patterns, population demography, and genetic parameters) can offer insights necessary to minimize real or perceived conflicts, but carnivores can be notoriously challenging to monitor and these challenges are amplified when surveying low-density or colonizing populations at the limit of a species’ range [20]. Camera-based surveys (i.e., camera trapping) [21] and noninvasive genetic sampling (NGS) [22] offer effective approaches for investigating low-density populations. Camera trapping is noninvasive, applicable in a variety of land-cover types, cost efficient, and facilitates broad-scale sampling [21]. Camera-based detection data are commonly analyzed within an occupancy modeling framework to associate patterns of occurrence with environmental factors while accounting for imperfect detection [23,24]. Cameras may also provide valuable auxiliary information such as evidence of reproduction [25]. NGS can facilitate individual and sex identification, as well as analyses of population genetic parameters [22,26]. When combined with capture-recapture models, NGS can be extended to estimate abundance [27,28].

American black bears (*Ursus americanus*) were once widespread across much of North America [29]. Black bears declined in abundance and distribution due to habitat loss and unregulated harvest, but have since expanded from remaining and translocated populations to occupy 65–75% of their historical range [29]. Black bear population declines were attributed, in part, to loss of forest cover and the species has often been considered a forest obligate [29]. Genetic analyses indicated that the Great Plains, an area with very little tree cover, constituted a significant barrier to gene flow between black bear populations in the Central Interior Highlands of the southeastern United States (primarily Arkansas and Missouri) and those in mountains of the southwestern United States [30]. Still, prior to broad-scale population declines, black bears historically occupied riparian (canopied) river corridors in the Great Plains [31], and these areas may have been critical to supporting gene flow from robust populations.

**Fig 1:**
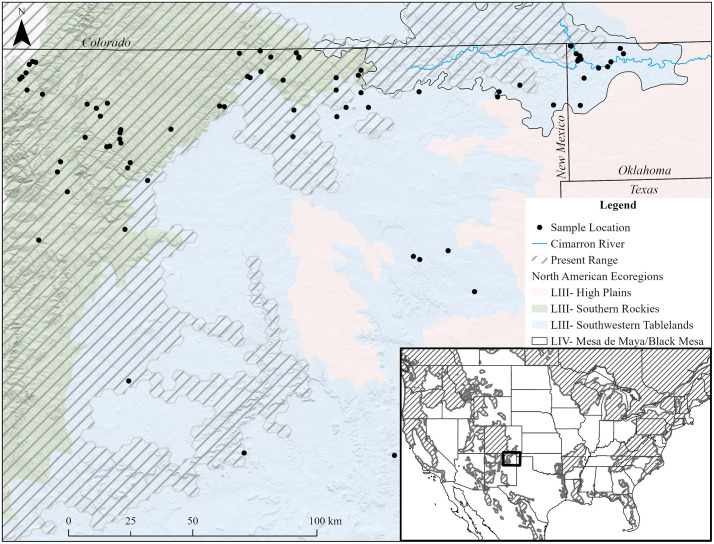
Distribution of the location of the first DNA sample collected per individual for black bears (*Ursus americanus*) in northern New Mexico and western Oklahoma in 2022 and 2023. Included are the Cimarron River, the predicted present range of black bear in North America, and selected level III ecoregions, with the Mesa de Maya/Black Mesa level IV ecoregion denoted. The predicted range of black bears was obtained from the International Union for Conservation of Nature (IUCN; reprinted from [29] under CC BY license, with permission from C. Hilton-Taylor [IUCN], original copyright [2016]), and the ecoregion boundaries were obtained from the U.S. Environmental Protection Agency (EPA) [32]. Elevational data were obtained from LANDFIRE [33], and political boundaries (available at https://www.cec.org/north-american-environmental-atlas/political-boundaries-2021/) and Cimarron River (available at https://www.cec.org/north-american-environmental-atlas/lakes-and-rivers-2023/) layers were obtained from the North American Environmental Atlas; these data are in the public domain.

Black bears have recently been documented in western Oklahoma—an area outside of the species’ historical range (Fig 1) [1]—in a sparsely forested region characterized by areas of semi-arid rangelands with canyons and mesas, and areas of relatively flat grasslands interspersed with croplands [32]. The density of nearby black bear populations in northern New Mexico and southern Colorado has reportedly increased over the early 21^st^ century [34,35]. The mean density of black bears in northern New Mexico was estimated to be approximately 17.0 bears per 100 km^2^ in 2001 [34] and increased to 21.9 bears per 100 km^2^ by 2018 [35]. Within New Mexico, the black bear population in northern New Mexico had the second highest density, closely following a region in central New Mexico that had a black bear density of 25.8 bears per 100 km^2^ [35]. Estimates of black bear densities from 2009–2015 in southern Colorado ranged from 21.0-44.0 bears per 100 km^2^, and were generally higher than density estimates throughout northern Colorado [36]. Thus, bears in western Oklahoma may represent animals dispersing along the Cimarron River from a high-density population, areas with low resource availability in northern New Mexico, or both [37].

Understanding the origin of black bears in western Oklahoma, their space use patterns, and the status of bears (i.e., transient individuals vs. a resident population) may provide insights into factors driving historical occurrence of black bears in unforested systems and along river corridors of the Great Plains. To this end, we sampled a presumably low-density black bear population on the species’ range extent using (i) cameras to evaluate factors associated with black bear space-use patterns and (ii) noninvasive genetic sampling to estimate population abundance and sex ratio. We also (iii) combined noninvasive genetic samples from western Oklahoma with genetic samples from bears harvested in New Mexico to assess population genetics parameters (i.e., genetic diversity and population structure). We hypothesized that high summer temperatures would constrain black bear space use to microhabitat features that reduced physiological stress [38] and, therefore, predicted space use would be positively associated with canopy cover. Black bears have tended to exhibit male-biased dispersal patterns [39], and we therefore predicted that bears in western Oklahoma would be predominantly male. Furthermore, mast producing oaks (*Quercus* spp.) important to black bears in other regions [34] were presumed to be relatively rare in western Oklahoma, which led us to predict that black bears would occur at relatively low abundance. We hypothesized that black bears in western Oklahoma would not be genetically differentiated from the genetic population distributed across northern New Mexico and southern Colorado [40], but that there would be evidence of male-biased dispersal [39].

## Materials and methods

### Study area

Our study encompassed portions of Cimarron County in western Oklahoma and northeastern New Mexico (Fig 1), which was predominantly characterized as Southwestern Tablelands straddled by High Plains to the east and Southern Rockies to the west (ecoregions) [32]. The Cimarron River traversed from the Sangre de Cristo Mountains in northeastern New Mexico to northwestern Oklahoma. Elevation ranged from ~3500 m in New Mexico to ~1200 m in Oklahoma [33]. In western Oklahoma, the warmest and coldest months on average were July (~26 °C) and January (~2 °C), respectively [41]. The average annual precipitation was ~ 466 mm with August being the wettest month (~82 mm) and February the driest (~9 mm) [41]. In northeast New Mexico, the warmest and coldest months on average were July (~21 °C) and December (−1 °C), respectively [42]. The average annual precipitation was ~ 349 mm with July being the wettest month (~57 mm) and February the driest (~8 mm) [42].

### Camera-based sampling

We overlaid the Oklahoma portion of our study area with a grid of 6.25-km^2^ cells in order to maximize coverage and detection of bears in the area, excluded cells for which access permission could not be obtained, and randomly selected 160 cells (hereafter, sites). Within each site, we set one motion-triggered Bushnell CORE™ DS-4K camera (Bushnell Corporation, Overland Park, KS) near features presumed to be important to bears (e.g., travel routes, thermal cover) with a scent pile containing one of four lures—skunk-based lure, beaver castor lure, blueberry lure, or anise oil—placed ~5 m in front of the camera (camera settings and lures are detailed in S1 Text). We deployed cameras during summer (May–August), with 80 sites being sampled during each of the two years (2022–2023). We deployed a camera at each site for ~28 days and checked cameras every 7–10 days to refresh memory cards, batteries, and lure. We processed images and identified species detected with Timelapse2 [43] and used a double review process to reduce misclassifications [44]. We required black bear images at the same site to be separated by ≥30 minutes to be considered independent detections [45]. We used black bear independent detections to generate a daily encounter history for each site indicating if a bear was detected (1) or not (0).

### Space-use covariates

We collected covariates hypothesized to influence black bear detection and space use. We hypothesized that black bear detection would be influenced by lure type and daily environmental conditions (i.e., temperature and precipitation), and would vary temporally (both within and between years) [46]. We characterized daily temperature (C°) and precipitation (mm) for each camera from spatially explicit raster datasets [47,48]. To account for temporal variation in detection within and between years, we included day of year and year as covariates. We hypothesized that black bear space use would be influenced by landcover and landscape features commonly associated with resource availability and movement. In arid systems, black bears have been documented supplementing their diet with cacti (particularly prickly pear [*Opuntia* spp.], which provides water), ants, and wasps [49,50]. Coarse woody debris (CWD) is commonly used by ants, wasps, other invertebrates, and small mammals [51]. Junipers (*Juniperus* spp.) are the dominant mast producing species in the region, and their prevalence on the landscape as a food resource may have direct implications on black bear reproduction [52]. Additionally, bears may select for areas with more thermal cover to escape the heat [53]. Elevation and slope can relate to water access [54], movement [55], or seasonal availability of food resources for black bears [56] which are all critical during the dry, hot summer months when water and food availability is diminished.

We characterized landcover and landscape features of each site using field-based sampling and remotely sensed data. Because fine-scale placement of cameras aimed to maximize the field of view and minimize false triggers (e.g., from wind-blown vegetation), we conducted field-based sampling by selecting a random location ≤ 30m from the camera, from which we established four 50-m transects; the bearing of the first transect was randomly selected and other transects were 90° from the previous transect. We counted the number of prickly pear cacti (separated by >0.25 m) and ant mounds within one meter of each transect, summing counts across transects to produce a single value per site. We quantified the relative amount of CWD that intercepted the transects by counting the number of downed logs with a diameter ≥10 cm [57] and brush piles with CWD and summing counts across transects to produce a single value per site. We estimated percent canopy cover at each site, with a spherical densiometer (Forestry Suppliers, Inc., Jackson, MS, USA), by averaging five measurements that we collected at the center of the sampling area and at the end of each transect. Finally, we recorded ocular estimates of percent landcover within 50 m attributable to juniper and oak (though oaks were not documented at any site). We collected broad-scale estimates of mean canopy cover (%) [58], slope (°) [59], and elevation (m) [33] at six different buffer distances (250–1,500 m, at 250-m intervals) around each camera using remotely sensed data (S2 Text).

We ensured our covariate set did not include any patterns of association or correlation. Repeat measures of a single covariate within nested buffers are inherently associated. For each broad-scale occupancy covariate measured at competing buffer sizes (i.e., scales), we evaluated relative support for each buffer using a model selection framework and retained only the most-supported buffer size for each covariate in subsequent analyses (S2 Text). We evaluated collinearity among covariates with Pearson’s correlation tests. Among comparisons, only two covariates—the most-supported buffer distances of slope (1,500 m) and canopy cover (1,500 m)—were correlated (|*r*| > 0.7). Model selection results indicated that broad-scale slope (within a 1,500 m buffer) was more supported by the data than broad-scale canopy cover (within a 1,500 m buffer), so we retained broad-scale slope and excluded broad-scale canopy cover from subsequent analyses (S2 Text).

### Space-use analyses

We analyzed black bear encounter histories using single-season, single-species occupancy models in Program MARK [23,60]. The scale of our sampling sites was small relative to the movement capacity of black bears, and we included sites sampled in each year into a single analysis. Consequently, we likely violated the closure assumption and interpreted occupancy (*ψ*) as reflecting the probability of use [61,62]. We developed a candidate model set for black bears by considering all additive combinations of uncorrelated covariates within each submodel (i.e., for detection [*p*] and *ψ*) and all combinations of submodels [63]. We used Akaike’s Information Criterion corrected for small sample size (AIC_c_) and differences in AIC_c_ (∆AIC_c_, where ∆_*i*_ = AIC_c*i*_ − AIC_c*min*_) to evaluate relative support for each model [64,65]. We evaluated the influence of covariates by considering the structure of the most-supported models, considering uninformative parameters, and assessing the direction and significance of beta coefficients with 85% confidence intervals [66,67]. We also considered cumulative predictor weights as an alternative approach to evaluate relative predictor importance while accounting for model selection uncertainty [66]; we considered weights ≥0.6 as strong support and weights ≥0.4 and <0.6 as moderate support [68]. To test for evidence of model assumption violations, we assessed goodness of fit for the global model using 1,000 parametric bootstrap replicates on a χ2 statistic appropriate for binary data (implemented in ‘unmarked’) [69–71].

### Genetic sampling

We anticipated bears to be rare in western Oklahoma, so we used camera-based detections to inform NGS. Following the detection of a black bear at a camera, we deployed a hair snare ≤15 m from the camera location and up to four additional hair snares ≥1.25 km away (the number deployed was constrained by landowner permissions). We constructed corral-style hair snares with one strand of barbed wire 45 cm above the ground mounted to ≥3 structures that were ~5–6 m apart [35]. We placed a scent pile in the center of the hair snare and randomly selected one of the four scent lures. We deployed hair snares for 28 days and checked every 7 days for hair samples to reduce DNA degradation [72]. We opportunistically collected hair samples from barbed-wire fences near sites. We collected hair samples with sterilized tweezers into paper coin envelopes and stored them in silica desiccant at −20 °C until DNA extraction [73]. We also opportunistically collected scat samples for DNA, placing 0.2 ml of fecal material from the side of scats in a tube with a DETS preservation buffer (i.e., 20% DMSO, 0.25 mol/L EDTA, 100 μmol/L Tris, pH 7.5, and NaCl to saturation) and stored at room temperature until DNA extraction [74,75]. To assess genetic connectivity with black bears in the nearest known population in the Southern Rockies ecoregion, we requested tissue samples from hunter-harvested black bears in northeastern New Mexico from the New Mexico Department of Game and Fish; black bears in northern New Mexico were part of a larger population including southern Colorado [40]. We dry stored tissue samples with silica desiccant at room temperature. Black bear sampling via cameras and hair snares was approved by the Institutional Animal Care and Use Committee at Oklahoma State University (protocol number IACUC-22–20).

### Genetic laboratory analyses

We extracted all samples using Zymo Quick-DNA extraction kits (Zymo Research, Irvine, CA USA) with noninvasive samples being extracted in a laboratory dedicated to low quality DNA samples with negative controls. For individual identification, we amplified black bear samples using a polymerase chain reaction (PCR) multiplex with primers for 11 nuclear DNA microsatellite loci and a sex identification primer within a single multiplex (S3 Text). We visualized PCR products on a 3130xl Genetic Analyzer (Applied Biosystems, Foster City, CA, USA) and scored allele sizes using Genemapper 5.0 (Applied Biosystems). PCR conditions including primer concentrations and thermal profiles are presented in supporting information (S3 Text). We conducted all laboratory procedures at the Laboratory for Ecological, Evolutionary and Conservation Genetics (University of Idaho, Moscow, ID USA).

We screened hair and fecal samples for amplification success using replicate PCRs via a multitubes approach [76] and established consensus genotypes by comparing replicates using ConGenR in Program R [77,78]. We amplified tissue and hair samples 2–4 times and fecal samples 4–6 times to obtain consensus genotypes. For tissue and hair samples, we required each allele to be observed ≥2 times across replicates before a consensus genotype was established for heterozygotes and homozygotes [79,80]. For fecal samples, we required each allele to be observed ≥2 times across replicates to establish a heterozygous consensus genotype and ≥3 times to establish a homozygous consensus genotype and avoid genotyping errors [77]. Allelic dropout errors occur when an allele in the consensus genotype fails to amplify in a replicate, whereas false allele errors are alleles appearing in a replicate that are not in the consensus genotype [81]. To minimize genotyping errors (i.e., allelic dropout and false alleles), we removed low-quality samples (amplification success of <40% across loci) [73]. We also dropped samples that failed to amplify at enough loci (7) to achieve a probability of identity for siblings <0.01 [82] (S3 Text). We aligned consensus genotypes to identify samples from the same individual and calculated genotyping error rates in ConGenR [77]. We evaluated the reliability of multilocus genotypes observed in only one sample with Reliotype [83], and we retained samples with a reliability of ≥95%.

### Genetic diversity and structure

Including related individuals in genetic diversity and population structure analyses may bias results or indicate false population structure [84, 85]. We estimated pairwise relatedness using the Queller and Goodnight [86] method with GenAlEx v6.51b2 [87,88], identified first-order relatives (i.e., relatedness values ≥0.45) [89], and parsimoniously removed one individual from each related pair [79]. We calculated observed heterozygosity (H_o_), unbiased expected heterozygosity (H_e_), and inbreeding coefficients (i.e., F_IS_) using GenAlEx, and allelic richness (A_r_) using FSTAT v2.9.4 [90]. We tested for departure from Hardy-Weinberg equilibrium (HWE) and linkage equilibrium for all loci across samples with the U-test for heterozygote deficiency and log-likelihood ratio statistic (G-test), respectively, and for population-wide departure from HWE with a global test of heterozygote deficiency using Genepop v4.7.5 [91] with default parameterizations (i.e., Dememorization = 1,000, Batches = 100, Iterations = 1,000) and Bonferroni corrections [92].

We used Bayesian clustering algorithms implemented in the program Structure v2.3.4 to evaluate population genetic structure [93]. Structure uses genotypes to infer the number of genetic clusters (*K*) that best meets expectations of HWE and linkage equilibrium. We initially performed 20 independent runs of the aspatial Structure model for each potential *K* (range = 1–8) with 50,000 burn-in and 250,000 Markov Chain Monte-Carlo iterations using an admixture model with correlated alleles [93,94]. Structure may not perform well for continuously distributed species with weak differentiation between clusters, but including sample locations as a prior may help in identifying genetic clusters when structure is weak [93,95,96]. Thus, we also ran a location-informed Structure model that considered user-identified groups based on sampling locations. We assigned individuals into three groups based on a combination of level III and level IV ecoregions where the sample was collected; for black bears sampled >1 time via hair snares in Oklahoma, we used the location of the first detection. We (i) grouped samples collected in the Southern Rockies (Level III ecoregion; Fig 1) into a single group, as this area was previously demonstrated to be one genetic cluster [40]. We separated samples collected in the Southwestern Tablelands (Level III ecoregion) into two groups: (ii) those from the Mesa de Maya/Black Mesa (Level IV ecoregion; which included samples from Oklahoma and the Cimarron River drainage) and (iii) those from other portions of the Southwestern Tablelands (Fig 1). We performed 20 independent runs of the location-informed Structure model using the same settings as the aspatial analyses. We intended to infer the most likely *K* from each analysis using a combination of the maximum mean log likelihood (i.e., L(*K*)) [93] and the second-order rate of change in L(*K*) (i.e., Δ*K*) [97], but Δ*K* cannot be calculated when *K* = 1 and we were therefore unable to use Δ*K* (described in Results). We analyzed L(*K*) at each *K* in CLUMPAK [98] and selected the *K* value with the maximum mean L(*K*).

To assess fine-scale, sex-specific spatial genetic structure we conducted spatial autocorrelation analyses and visualized results with autocorrelograms (hereafter, correlograms) for male and female bears in GenAlEx. Correlograms evaluate relationships between geographic location and an autocorrelation coefficient (*r*_*a*_), where significant positive autocorrelation coefficients indicate fine-scale genetic structure is present [99]. Results of spatial autocorrelation can be influenced by distance class (i.e., bin) sizes and the interplay between the size (and number) of bins and the number of samples within each bin [99,100]. To select the appropriate bin size, we evaluated the influence of bin size on our results using an exploratory multiple-distance-class analysis with 999 permutations and 1,000 bootstrap samples to assess significance within each bin; we considered *r*_*a*_ values significant if the *r*_*a*_ value fell outside of the 95% confidence bounds or if the 95% bootstrap confidence interval did not overlap zero [100]. We used the multiple-distance-class analysis to select the smallest bin size with evidence of structure for either sex (i.e., 10 km; see Results), and then conducted sex-specific analyses with 25 even-distance classes of 10 km (0–250 km) with 9,999 permutations and 10,000 bootstraps. We analyzed the overall heterogeneity of each correlogram using ‘Omega’ (ω) [99,101] and considered test results significant when *P* ≤ 0.01, which is the recommend significance level when evaluating sex-biased dispersals to reduce erroneous results when there is some level of structure already identified within the population [99].

### Population demography

We used the number of unique multilocus genotypes that were either observed more than once (i.e., animals detected multiple times) or were determined to be reliable as the minimum count of black bears in the Oklahoma portion of our sampling extent. We estimated the ratio of males to females in Oklahoma based on sex identification results for bears included in the minimum black bear count. We also employed capture-with-replacement (CAPWIRE) models to estimate the abundance of black bears in our western Oklahoma sampling extent, while accounting for capture probabilities <1. CAPWIRE was developed specifically for NGS and uses repeat captures of individuals, including those within a single sampling event, to characterize capture probability and estimate abundance [27]. Equal effort across sites is an assumption of CAPWIRE models [27] and we therefore restricted analyses to hair samples. We assumed that the population was demographically closed within a sampling season and initially restricted CAPWIRE analyses to samples collected in 2022. We fitted models using both the equal capture (ECM) and two-innate rate capture (TIRM) models; we compared model fit using likelihood-ratio tests and generated 95% confidence intervals using parametric bootstrapping with 1,000 bootstraps [27]. We subsequently conducted a CAPWIRE analysis with sampling data from both 2022 and 2023 following the same procedures.

## Results

### Camera-based sampling and space use analyses

**Fig 2:**
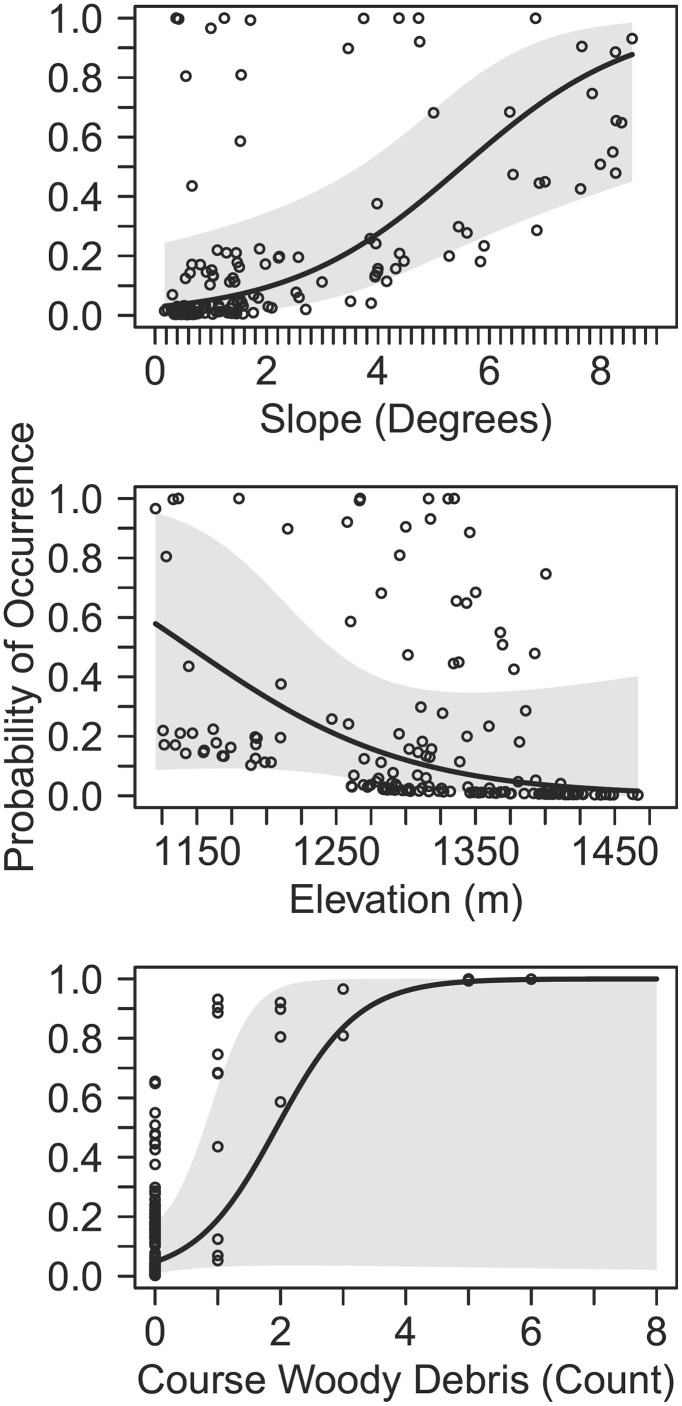
Estimated probability of occurrence (ψ) and data points (black dots) with 95% confidence intervals as a function of slope (top), elevation (middle), and coarse woody debris count (bottom) when other covariates were held at their mean values from the most-supported single-season single-species occupancy model for black bears (*Ursus americanus*) in Cimarron County, Oklahoma in 2022 and 2023.

We deployed cameras at 160 sites for a mean of 28.83 days (SD = 1.5 days). We documented 170 independent black bear detections with ≥1 black bear detection at 20 sites (naïve occupancy = 12.5%); detections included evidence of reproduction via detections of two different females with cubs at two sites. We did not find evidence for lack of fit based on the χ2 statistics (*P* = 0.864). Our final model set from the all-possible combinations approach included 2,048 models. The most-supported detection model indicated that black bear detection was positively associated with temperature and precipitation, negatively associated with day of year, and differed between years (Table 1, Table 2). When considering the full model set, cumulative predictor weights provided strong support for the effect of year on detection and moderate support for the effects of temperature, precipitation, and day of year (Table 2). The most-supported occupancy model indicated that black bear space use was negatively associated with elevation and positively associated with slope and CWD counts (Fig 2). Cumulative predictor weights provided strong support for the effects of CWD and slope, but only moderate support for the effect of elevation (Table 2).

**Table 1:** Results for single-season single-species occupancy analysis for detection (*p*) and space use (*ψ*) for black bears (*Ursus americanus*) in Cimarron County, Oklahoma, USA from 2022 to 2023, with models ranked based on Akaike’s information criterion with small sample size correction (AIC_c_) and difference in AIC_c_ (ΔAIC_c_), and reported with number of parameters (*K*), Akaike weight (*w*_i_), and log-likelihood (LL); only models within 2 ΔAIC_c_ of most-supported model and the null model are reported.

Detection Model	Space Use Model	*K*	AIC_c_	ΔAIC_c_	*w*_*i*_	LL
*p*(DOY+Year+Temp+Prec)	*ψ*(Elev+Slope+CWD)	9	401.80	0.00	0.03	382.60
*p*(Year)	*ψ*(Elev+Slope+CWD)	6	401.89	0.09	0.03	389.34
*p*(DOY+Year+Temp+Prec)	*ψ*(Slope+CWD)	8	401.93	0.13	0.03	384.97
*p*(Year)	*ψ*(Slope+CWD)	5	401.95	0.16	0.03	391.56
*p*(DOY+Year)	*ψ*(Slope+CWD)	6	402.64	0.85	0.02	390.09
*p*(Year+Temp+Prec)	*ψ*(Elev+Slope+CWD)	8	402.76	0.96	0.02	385.81
*p*(DOY+Year+Temp)	*ψ*(Slope+CWD)	7	402.81	1.01	0.02	388.07
*p*(Year+Prec)	*ψ*(Elev+Slope+CWD)	7	402.84	1.04	0.02	388.10
*p*(DOY+Year)	*ψ*(Elev+Slope+CWD)	7	402.89	1.10	0.02	388.16
*p*(Year+Prec)	*ψ*(Slope+CWD)	6	402.99	1.20	0.02	390.44
*p*(DOY+Year+Temp)	*ψ*(Elev+Slope+CWD)	8	403.08	1.28	0.02	386.12
*p*(Year+Temp+Prec)	*ψ*(Slope+CWD)	7	403.16	1.36	0.02	388.42
*p*(Year+Temp)	*ψ*(Elev+Slope+CWD)	7	403.20	1.40	0.02	388.46
*p*(Year+Temp)	*ψ*(Slope+PPC + CWD)	6	403.30	1.50	0.02	390.75
*p*(Year+Temp)	*ψ*(Slope+CWD+)	6	403.31	1.52	0.01	390.77
*p*(Year)	*ψ*(Elev+Slope+CWD+Junip)	7	403.74	1.94	0.01	389.00
*p*(DOY+Year+Prec)	*ψ*(Slope+CWD)	7	403.74	1.95	0.01	389.01
*p*(.)	*ψ*(.)	2	447.71	45.91	0.00	443.63

Note: Temp = Temperature (°C), DOY = Day of year, Prec = Precipitation (mm), CWD = Coarse woody debris count, Slope = Slope (°), Elev = Elevation (m), Junip = field collected percent juniper (*Juniperus* spp.) cover, PPC = Prickly pear cactus (*Opuntia* spp.) count, “.” indicates the intercept-only (i.e., null) model.

**Table 2:** Results of single-season single-species occupancy analysis for black bears (*Ursus americanus*) sampled in western Oklahoma (USA) during 2022–2023 including beta estimates (β), standard errors (SE), and 85% lower (LCL) and upper (UCL) confidence limits for covariates included in the most-supported model for detection (*p*) and space use (*ψ*), and cumulative predictor weights (Weight) for all covariates considered in the candidate model set (i.e., calculated using all models in the candidate model set).

Parameters	β	SE	LCL	UCL	Weight
*p* intercept	−1.064	1.895	−3.793	1.665	
Year	−2.234	0.824	−3.421	−1.048	0.979
Temp	0.079	0.040	0.023	0.136	0.509
DOY	−0.023	0.013	−0.041	−0.004	0.490
Prec	0.058	0.028	0.018	0.097	0.477
Lures	–	–	–	–	0.076
*ψ* intercept	12.456	10.586	−2.788	27.701	
CWD	1.539	0.851	0.314	2.765	0.997
Slope	0.645	0.236	0.305	0.984	0.891
Elev	−0.013	0.009	−0.025	0.000	0.498
Juniper	–	–	–	–	0.300
PPC	–	–	–	–	0.275
AM	–	–	–	–	0.272

Note: Temp = Temperature (°C); DOY = Day of year; Precip = Precipitation (mm); CWD = Coarse woody debris count; Slope = Slope (°); Elev = Elevation (m); Juniper = field collected percent juniper (*Juniperus* spp.) cover; PPC = Prickly pear cactus (*Opuntia* spp.) count; AM = Ant mound count; “-“ indicates the parameter was not included in the most-supported model.

### Genetic sampling and standard genetic measures

We collected 283 genetic samples. We deployed 41 hair snares (mean days per deployment = 26.9 ± 2.7 SD) and collected 153 hair samples (mean samples per snare = 3.7 ± 4.2 SD). We opportunistically collected 28 hair samples and 33 scat samples. We identified 43 of 181 hair samples and 14 of 33 scat samples as black bear. Allelic dropout rates (18.82%) were ~ 3.3x higher than false allele rates (5.65%). We achieved complete consensus genotypes across loci for 14 individuals; the remaining 7 individuals had a consensus genotype at an average of 9 (± 0.36 SE) loci (range = 8–10). Hair and fecal samples identified 21 unique black bears (12M:9F; ratio = 1.33:1) in the Oklahoma portion of our sampling extent. We obtained tissue samples from 69 black bears harvested in New Mexico (40M:29F; ratio = 1.34:1). We achieved complete consensus genotypes across loci for 57 individuals, and consensus genotypes at an average of 9 (±0.59 SE) loci (range = 3–10) for 12 individuals. We removed one New Mexico sample due to insufficient amplification among loci to achieve a probability of identity for siblings <0.01, resulting in 68 (39M:29F) individuals being retained for analysis.

Among 90 unique black bears sampled in New Mexico and Oklahoma, we removed 28 individuals (24 from New Mexico and 4 from Oklahoma) from genetic analyses to exclude first-order relationships. Across loci, the mean number of alleles was similar to the allelic richness (Table 3). Mean F_IS_ was not different from zero and indicated no substantial departure from random mating (Table 3). We found no evidence of departures from HWE across loci following Bonferroni corrections (Table 3), but we detected significant population-level heterozygote deficiency (*P* = 0.013). We found no evidence of departures in linkage equilibrium across loci.

**Table 3:** Sample size (N), number of alleles (N_a_), allelic richness (A_r_), observed heterozygosity (H_o_), unbiased expected heterozygosity (H_e_), fixation index (F_IS_), and *P*-value for tests of departure from Hardy-Weinberg Equilibrium (HWE) for 11 microsatellite loci for black bear (*Ursus americanus*) genetic samples in northern New Mexico and western Oklahoma in 2022 and 2023.

Loci	N	N_a_	A_R_	H_o_	H_e_	F_IS_	*P*-value
CXX20	57	7	6.89	0.632	0.610	−0.044	0.102
D1a	61	4	4.00	0.361	0.389	0.065	0.302
G10B	60	5	5.00	0.467	0.677	0.305	0.009
G10C*	61	3	3.00	0.557	0.464	−0.211	0.440
G10H	58	9	9.00	0.724	0.748	0.023	0.799
G10L	61	8	7.87	0.787	0.824	0.037	0.545
G10M	61	4	3.89	0.705	0.643	−0.105	0.914
G10X	61	5	4.89	0.770	0.712	−0.091	0.222
G1D	54	7	7.00	0.778	0.779	−0.008	0.407
Mu15	60	4	3.90	0.617	0.617	−0.008	0.467
Mu59	56	6	6.00	0.482	0.551	0.117	0.034
Mean	59.1	5.6	5.58	0.625	0.638	0.007	
*SE*	0.74	0.58	0.58	0.043	0.040	0.040	

Note: * = A_r_ estimated with 61 diploid individuals (compared to 54 diploid individuals used for other loci).

### Genetic structure analyses

**Fig 3:**
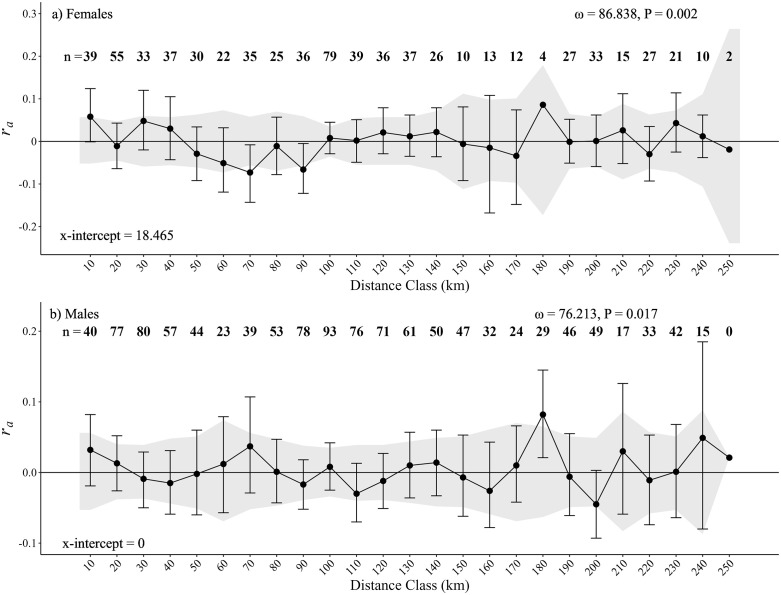
Correlograms showing autocorrelation coefficient (*r*_*a*_) at 10 km even distance classes including: 95% confidence bounds about the null hypothesis of no structure (*r*_*a*_ = 0, gray shading), 95% confidence intervals of *r*_*a*_ estimates (black dot) determined by bootstrap resampling, sample size (n), heterogeneity value ‘Omega’ (ω) and associated p-value (*P*), and x-intercept for female (a) and male (b) black bears (*Ursus americanus*) in northern New Mexico and western Oklahoma in 2022 and 2023 calculated in GenAlEx. Note that x-intercepts were considered zero when *r*_*a*_ was not significant.

The maximum mean L(*K*) from the aspatial STRUCTURE analyses indicated evidence for a single population (*K* = 1; S1 Fig). Considering the ecoregion where each black bear occurred with a location-informed STRUCTURE model did not elucidate any additional (e.g., cryptic) structure, with *K* = 1 still having the maximum mean L(*K*) (S1 Fig). Results of the multiple-distance-class analysis failed to detect any evidence of structure for males, but detected evidence of structure for females starting at a distance of 10 km (S2 Fig). Even-distance (10-km) bin analyses indicated positive genetic structure for females at 10 km with an x-intercept of 18.5 and an overall significant correlogram (ω = 86.838, *P* = 0.002; Fig 3). The overall 10-km bin correlogram for males was not significant (ω = 76.213, *P* = 0.017; Fig 3).

### Abundance estimation

When considering only hair samples from the 21 black bears detected in Oklahoma, individuals were captured 1–7 times (mean = 1.95 ± SD 1.82). Considering 2022 data, the ECM estimated 21 (95% CI = 17–27) black bears in the Oklahoma portion of our sampling extent, whereas the TIRM estimated 26 (95% CI = 18–43) black bears. Although the likelihood-ratio test failed to reject the ECM for 2022 (*P* = 0.47), likelihood ratio tests may fail to reject the ECM when sample sizes are small (as in our case) and/or when capture heterogeneity is present [26]. We expected, based on the results of our occupancy analyses, that capture probabilities were significantly different between years. When considering 2022 and 2023 data together, the likelihood-ratio test rejected the ECM (*P* < 0.001). Consequently, the TIRM model estimated 26 (95% CI = 19–43) black bears in the Oklahoma portion of our sampling extent during 2022–2023.

## Discussion

We investigated space use patterns, abundance, and genetic structure of black bears in a region that was outside of the historical range of the species and represented a distributional expansion into presumably low-quality habitat. Collectively, our estimates of black bear abundance, observed sex ratio, evidence of reproduction, and genetic analyses suggest that the occurrence of bears in western Oklahoma was likely a consequence of demographic expansion of the population in northern New Mexico and southern Colorado [40]. Results of our space-use analyses indicated black bears in western Oklahoma may be selecting for areas with adequate cover and forage. Studying species at the edge of their range extent may help characterize the species’ environmental limits and improve our understanding of their ability to persist under changing environmental conditions [102–104].

In our western Oklahoma study extent, areas characterized by low elevations and steep slopes tended to be associated with mesic or hydrologically ephemeral areas. Most of our black bear detections occurred near the Cimarron River—which produced deep canyons through the region extending into northern New Mexico [105,106]—and black bears may have been associated with low elevations and steep slopes in this area because they produced riparian areas [51]. Black bears have been associated with steeper slope and riparian areas at high-elevation mountainous sites in the western United States [107], but selection for these features may be more important in regions with warmer conditions. For example, black bear activity [108] and microhabitat selection for shade [53,109] may be influenced by high temperatures. We did not find support for our prediction that black bear space use would be associated with canopy cover, but patterns of association with elevation and slope may be indirectly related to thermal cover associated with riparian vegetation. In the northern Chihuahuan Desert, black bears tended to be associated with rugged terrain and brushy habitats that provided cover [55], similar to conditions along the Cimarron River. Vegetation occurring near the Cimarron River, which generally consisted of salt cedars (*Tamarix* spp.) and cottonwoods (*Populus* spp.) [106], may have provided thermal cover for bears. Bears may also use water sources for thermal regulation, particularly when temperatures exceed 24 °C [110]. We did not detect black bears in the High Plains ecoregion surrounding the Cimmaron River corridor; the High Plains in our study area was characterized primarily by shortgrass prairie and cropland [111] and likely failed to provide adequate concealment cover important to black bears [112]. Thus, black bears historically documented along rivers in the Great Plains [31] may have also been limited by thermal or concealment cover outside of the mesic conditions provided along river corridors.

Black bears can exploit a wide range of food resources and in the southwestern United States have relied on soft mast (e.g., berries), hard mast (e.g., acorns), ants, yucca (*Yucca* spp.), and prickly pear cacti [35,52,55,113]. We found no evidence that black bear space use was influenced by indices of mast producing trees, ant mounds, or prickly pear presence. Relative CWD was not correlated with canopy cover in our system, but the positive association we observed between bear space use and relative CWD may be related to food availability. Coarse woody debris supports invertebrates and small mammals and, thus, may influence black bear foraging [114,115]. Raine & Kansas [56] found that bears in Canada were associated with logs, which were related to the presence of ants or ant larvae. In arid systems, flow regimes of intermittent or ephemeral waterways can transport debris and create piles that support high arthropod richness [116,117]. Although relative CWD was the only food-related covariate that appeared to influence space use patterns of black bears, our sampling period included only two summers and black bear foraging behavior may vary among years as a consequence of dynamic food resources. For example, bears in Colorado undulated between using wildland areas more in years of good natural resource availability, and using more urban areas more during years of poor natural resource availability [118]. Reduced precipitation can negatively influence food availability for black bears (e.g., mast crops) and, even in temperate forests, human-bear conflicts had an inverse relationship with precipitation [119]. Predicting periods of drought may be critical to predicting the spatial availability of forage, associated space-use patterns of black bears, and the potential for human-bear interactions. In years of poor natural food availability, bears may attempt to supplement their diet with anthropogenic food sources, which increases the potential for human-black bear interactions [120]. For example, human-black bear interactions occurred almost 5 × more often during La Niña years than El Niño years in New Mexico [121]. Similarly, a severe drought diminished natural resources near Lake Tahoe (California-Nevada), leading to a substantial increase in human-bear conflicts compared to prior years [122]).

Genetic analyses indicated black bears occurring in western Oklahoma were part of a larger population distributed across much of northern New Mexico and southern Colorado (i.e., the Sangre de Cristo Mountain population) [40]. Black bear densities in New Mexico have reportedly been relatively stable (and potentially increasing) [35] and the observed population-level heterozygote deficiency and no linkage disequilibrium were consistent with patterns expected in a growing population (i.e., demographic expansion) [123]. The Cimmaron River corridor extends from the eastern portion of the Sangre de Cristo Mountains into northwestern Oklahoma and likely facilitated the expansion of black bears. Black bears may disperse further from higher-density populations [39]. In New Mexico, black bears tended to exhibit male-biased dispersal patterns with female dispersal being more limited (typically <20 km) [39,124]. Our spatial autocorrelation analyses corroborated these patterns, providing evidence for male-biased dispersal and suggesting that female dispersal was typically limited to <20 km (x-intercept = 18.5 km for the females) [125]. The sex ratio of black bears sampled in Oklahoma via hair snares and fecal samples (1.33M:1F) was similar to the sex ratio of harvested bears in northern New Mexico (1.34M:1F). Nonetheless, black bear harvest may be male-biased if hunters target larger bears [126–128]. Our observed sex ratios from both harvested bears in New Mexico and those sampled via hair snares in Oklahoma were more male biased than sex ratios estimated from hair snares in the Sangre De Cristo Mountains (northern portion = 1.01M:1F, southern portion = 1.05M:1F) [35].

Despite the apparent marginal male bias in the black bear population in western Oklahoma, estimates of abundance and evidence of reproduction (i.e., detections of females with cubs) indicate that black bears have become established and the habitat is sufficient to support reproduction [129]. Costello [130] reported larger median 95% home range sizes for males (~463 km^2^) than females (~100 km^2^) in New Mexico and based on these estimates our full Oklahoma sampling extent would be expected to support ~10 male and ~48 female non-overlapping home ranges. Conversely, the portion of our sampling extent that aligned with space-use patterns of black bears (i.e., Mesa de Maya/Black Mesa level IV ecoregion) would be expected to support fewer non-overlapping home ranges (~2 male and ~9 female). Although these rough estimates do not account for overlapping home ranges, which is common between female relatives [124,131,132], they are based on home range size estimates from areas in the core of the species distribution, where the habitat quality is presumably greater than in our study area, which is outside of the historical distribution and lacking important food resources found elsewhere (e.g., oak mast). Future monitoring to assess population stability may provide greater insights into factors limiting black bears in this region and at the species range limit.

Even during periods of climate variability, species with good dispersal and colonization abilities can successfully expand their range in areas of habitat fragmentation [133]. Movement of individuals from the interior of the species range to the edge of the range maintain gene flow, which can increase genetic variation and fitness [134,135], and these benefits may be even more pronounced when gene flow occurs between individuals experiencing similar environmental conditions within the range [136]. Droughts are increasing in frequency and intensity throughout the Great Plains, including western Oklahoma [137], and low genetic diversity may limit a species’ ability to respond to these environmental changes [138]. Investigating the ecology of species at the leading edge of a range expansion can provide insights into factors limiting population distribution, abundance, and genetic health, and may elucidate how species will respond to future environmental changes. For large carnivores, understanding factors promoting or limiting space use and abundance can facilitate proactive management to minimize human-wildlife conflicts or mitigate conflicts that arise [20,107].

## Supporting information

S1 Fig Population genetic clustering results. (PDF)

S1 Text Details for remote camera settings and scent lure selection. (PDF)

S2 Fig Results of multiple-distance-class analyses. (PDF)

S2 Text Analysis methods and results comparing support for competing buffer widths for covariates. (PDF)

S3 Text DNA extraction and PCR conditions. (PDF)

## References

[1] Scheick BK, McCown W. Geographic distribution of American black bears in North America. Ursus. 2014;25(1):24. doi: 10.2192/ursus-d-12-00020.1

[2] Chen I-C, Hill JK, Ohlemüller R, Roy DB, Thomas CD. Rapid range shifts of species associated with high levels of climate warming. Science. 2011;333(6045):1024–6. doi: 10.1126/science.1206432 21852500

[3] Wait KR, Ahlers AA. Virginia opossum distributions are influenced by human-modified landscapes and water availability in tallgrass prairies. J Mammal. 2020;101(1):216–25.

[4] Beckmann JP, Berger J. Using black bears to test ideal-free distribution models experimentally. J Mammal. 2003;84(2):594–606.

[5] Ditmer MA, Noyce KV, Fieberg JR, Garshelis DL. Delineating the ecological and geographic edge of an opportunist: the American black bear exploiting an agricultural landscape. Ecol Model. 2018;387:205–19.

[6] Lyons SK. A quantitative assessment of the range shifts of Pleistocene mammals. J Mammal. 2003;84(2):385–402.

[7] Buckley LB, Kingsolver JG. Functional and phylogenetic approaches to forecasting species’ responses to climate change. Annu Rev Ecol Evol Syst. 2012;43(1):205–26.

[8] Pacifici M, Rondinini C, Rhodes JR, Burbidge AA, Cristiano A, Watson JEM, et al. Global correlates of range contractions and expansions in terrestrial mammals. Nat Commun. 2020;11(1):2840. doi: 10.1038/s41467-020-16684-w 32504033 PMC7275054

[9] Beissinger SR, Riddell EA. Why Are Species’ Traits weak predictors of range shifts?. Annu Rev Ecol Evol Syst. 2021;52(1):47–66. doi: 10.1146/annurev-ecolsys-012021-092849

[10] Blois JL, Zarnetske PL, Fitzpatrick MC, Finnegan S. Climate change and the past, present, and future of biotic interactions. Science. 2013;341(6145):499–504. doi: 10.1126/science.1237184 23908227

[11] Wolf C, Ripple WJ. Range contractions of the world’s large carnivores. R Soc Open Sci. 2017;4(7):170052. doi: 10.1098/rsos.170052 28791136 PMC5541531

[12] Pratzer M, Nill L, Kuemmerle T, Zurell D, Fandos G. Large carnivore range expansion in Iberia in relation to different scenarios of permeability of human‐dominated landscapes. Diversity and Distributions. 2022;29:75–88.

[13] Lanszki J, Hayward MW, Ranc N, Zalewski A. Dietary flexibility promotes range expansion: the case of golden jackals in Eurasia. J Biogeogr. 2022;49(6):993–1005.

[14] Cimatti M, Ranc N, Benítez‐López A, Maiorano L, Boitani L, Cagnacci F. Large carnivore expansion in Europe is associated with human population density and land cover changes. Diversity and Distributions. 2021;27(4):602–17.

[15] Ditmer MA, Garshelis DL, Noyce KV, Haveles AW, Fieberg JR. Are American black bears in an agricultural landscape being sustained by crops?. J Mammal. 2016;97(1):54–67.

[16] Chapron G, Kaczensky P, Linnell JDC, von Arx M, Huber D, Andrén H, et al. Recovery of large carnivores in Europe’s modern human-dominated landscapes. Science. 2014;346(6216):1517–9. doi: 10.1126/science.1257553 25525247

[17] Unger D, Cox J, Harris H, Larkin J, Augustine B, Dobey S. History and current status of the black bear in Kentucky. Northeastern Naturalist. 2013;20:289–308.

[18] Ripple WJ, Estes JA, Beschta RL, Wilmers CC, Ritchie EG, Hebblewhite M, et al. Status and ecological effects of the world’s largest carnivores. Science. 2014;343(6167):1241484. doi: 10.1126/science.1241484 24408439

[19] Treves A, Karanth KU. Human-carnivore conflict and perspectives on carnivore management worldwide. Conserv Biol. 2003;17(6):1491–9.

[20] Frary VJ, Duchamp J, Maehr DS, Larkin JL. Density and distribution of a colonizing front of the American black bear Ursus americanus. Wildlife Biology. 2011;17(4):404–16. doi: 10.2981/09-103

[21] Burton AC, Neilson E, Moreira D, Ladle A, Steenweg R, Fisher JT. Wildlife camera trapping: A review and recommendations for linking surveys to ecological processes. J Appl Ecol. 2015;52(3):675–85.

[22] Waits LP, Paetkau D. Noninvasive genetic sampling tools for wildlife biologists: A review of applications and recommendations for accurate data collection. J Wildl Manage. 2005;69(4).

[23] MacKenzie DI, Nichols JD, Lachman GB, Droege S, Andrew Royle J, Langtimm CA. ESTIMATING SITE OCCUPANCY RATES WHEN DETECTION PROBABILITIES ARE LESS THAN ONE. Ecology. 2002;83(8):2248–55. doi: 10.1890/0012-9658(2002)083[2248:esorwd]2.0.co;2

[24] MacKenzie D, Nichols JD, Royle JA, Pollock K, Bailey LL, Hines J. Occupancy estimation and modeling: inferring patterns and dynamics of species occurrence. 2nd ed. San Diego: Academic Press. 2018.

[25] Fisher JT, Wheatley M, Mackenzie D. Spatial patterns of breeding success of grizzly bears derived from hierarchical multistate models. Conserv Biol. 2014;28(5):1249–59. doi: 10.1111/cobi.12302 24762089

[26] Schwartz MK, Luikart G, Waples RS. Genetic monitoring as a promising tool for conservation and management. Trends Ecol Evol. 2007;22(1):25–33. doi: 10.1016/j.tree.2006.08.009 16962204

[27] Miller CR, Joyce P, Waits LP. A new method for estimating the size of small populations from genetic mark-recapture data. Mol Ecol. 2005;14(7):1991–2005. doi: 10.1111/j.1365-294X.2005.02577.x 15910321

[28] Lonsinger RC, Lukacs PM, Gese EM, Knight RN, Waits LP. Estimating densities for sympatric kit foxes (Vulpes macrotis) and coyotes (Canis latrans) using noninvasive genetic sampling. Can J Zool. 2018;96(10):1080–9.

[29] Garshelis DL, Scheick BK, Doan-Crider DL, Beecham JJ, Obbard ME. Ursus americanus. The IUCN Red List of Threatened Species. https://www.iucnredlist.org/species/41687/114251609. 2016. Accessed 2024 May 1.

[30] Puckett EE, Etter PD, Johnson EA, Eggert LS. Phylogeographic Analyses of American Black Bears (Ursus americanus) Suggest Four Glacial Refugia and Complex Patterns of Postglacial Admixture. Mol Biol Evol. 2015;32(9):2338–50. doi: 10.1093/molbev/msv114 25989983

[31] LALIBERTE AS, RIPPLE WJ. Wildlife Encounters by Lewis and Clark: A Spatial Analysis of Interactions between Native Americans and Wildlife. BioScience. 2003;53(10):994. doi: 10.1641/0006-3568(2003)053[0994:weblac]2.0.co;2

[32] Level III ecoregions of the continental United States. https://www.epa.gov/eco-research/level-iii-and-iv-ecoregions-continental-united-states. 2013. Accessed 2024 December 15.

[33] Elevation Layer. http://www.landfire/viewer. 2022. Accessed 2023 September.

[34] Costello CM, Jones DE, Hammond K, Inman RM, Inman K, Thompson BC. A study of black bear ecology in New Mexico with models for population dynamics and habitat suitability: Federal Aid in Wildlife Restoration Project W-131-R Final Report. 2001.

[35] Gould MJ, Cain III JW, Roemer GW, Gould WR, Liley SG. Density of American black bears in New Mexico. J Wildl Manage. 2018;82(4):775–88.

[36] Apker JA, Runge J, Johnson H, Mao J, Vitt A, Vieira M, et al. Non-invasive genetic-based black bear investigations in Colorado—2009–2015. Monte Vista, USA: Colorado Division of Wildlife. 2016.

[37] Kopsala E, Kyle C, Howe E, Potter D, Beauclerc K, Northrup JM. Broad‐scale genetic monitoring suggests density‐dependent dispersal in a large carnivore. Oikos. 2023;2023(7). doi: 10.1111/oik.09442

[38] Riddell EA, Iknayan KJ, Hargrove L, Tremor S, Patton JL, Ramirez R, et al. Exposure to climate change drives stability or collapse of desert mammal and bird communities. Science. 2021;371(6529):633–6. doi: 10.1126/science.abd4605 33542137

[39] Costello CM, Creel SR, Kalinowski ST, Vu NV, Quigley HB. Sex-biased natal dispersal and inbreeding avoidance in American black bears as revealed by spatial genetic analyses. Mol Ecol. 2008;17(21):4713–23. doi: 10.1111/j.1365-294X.2008.03930.x 18828781

[40] Gould MJ, Cain JW 3rd, Atwood TC, Harding LE, Johnson HE, Onorato DP, et al. Pleistocene-Holocene vicariance, not Anthropocene landscape change, explains the genetic structure of American black bear (Ursus americanus) populations in the American Southwest and northern Mexico. Ecol Evol. 2022;12(10):e9406. doi: 10.1002/ece3.9406 36248671 PMC9551525

[41] U.S. Climate Normals Quick Access (1991-2021) for Boise City 2 E, Oklahoma. https://www.ncei.noaa.gov/access/us-climate-normals/#dataset=normals-annualseasonal&timeframe=30&station=USC00340908. 2024. Accessed 2024 March.

[42] U.S. Climate Normals Quick Access (1991-2021) for Raton Municipal/Crews Field Airport, New Mexico. Asheville, NC: National Oceanic and Atmospheric Administration [NOAA]. 2024. https://www.ncei.noaa.gov/access/us-climate-normals/#dataset=normals-annualseasonal&timeframe=30&station=USW00023052

[43] Greenberg S, Godin T, Whittington J. Design patterns for wildlife-related camera trap image analysis. Ecol Evol. 2019;9(24):13706–30. doi: 10.1002/ece3.5767 31938476 PMC6953665

[44] Lonsinger RC, Dart MM, Larsen RT, Knight RN. Efficacy of machine learning image classification for automated occupancy‐based monitoring. Remote Sens Ecol Conserv. 2023;10(1):56–71. doi: 10.1002/rse2.356

[45] Iannarilli F, Erb J, Arnold TW, Fieberg JR. Evaluating species-specific responses to camera-trap survey designs. Wildlife Biology. 2021;2021(1). doi: 10.2981/wlb.00726

[46] Sollmann R. Mt or not Mt: Temporal variation in detection probability in spatial capture-recapture and occupancy models. Peer Community Journal. 2024;4. doi: 10.24072/pcjournal.357

[47] McEnery J, Ingram J, Duan Q, Adams T, Anderson L. NOAA’s Advanced Hydrologic Prediction Service: Building pathways for better science in water forecasting. Bull Amer Meteor Soc. 2005;86(3):375–86.

[48] Recent years (Jan 1981–Aug 2023). Corvallis (OR): PRISM Climate Group. 2023. https://prism.oregonstate.edu

[49] Corn JL, Warren RJ. Seasonal food habits of the collared peccary in south texas. J Mammal. 1985;66(1):155–9.

[50] McClinton SF, McClinton PL, Richerson JV. Food habits of black bears in Big Bend National Park. Southwest Nat. 1992;37(4).

[51] Loeb SC. The role of coarse woody debris in the ecology of southeastern mammals. Washington, DC: US Forest Service. 1993.

[52] Costello CM, Jones DE, Inman RM, Inman KH, Thompson BC, Quigley HB. Relationship of variable mast production to American black bear reproductive parameters in New Mexico. Ursus. 2003;14(1):1–16.

[53] Pigeon KE, Cardinal E, Stenhouse GB, Côté SD. Staying cool in a changing landscape: the influence of maximum daily ambient temperature on grizzly bear habitat selection. Oecologia. 2016;181(4):1101–16. doi: 10.1007/s00442-016-3630-5 27085998

[54] Rice MB, Ballard WB, Fish EB, McIntyre NE, Holdermann D. Habitat-Distribution Modeling of a Recolonizing Black Bear, *Ursus americanus*, Population in the trans-pecos region of Texas. Can Field Nat. 2009;123(3):246. doi: 10.22621/cfn.v123i3.972

[55] Hellgren EC. Status, distribution, and summer food habits of black bears in Big Bend National Park. Southwest Nat. 1993;38(1):77–80.

[56] Raine RM, Kansas JL. Black Bear Seasonal Food Habits and Distribution by Elevation in Banff National Park, Alberta. Bears: Their Biol Manag. 1990;8:297. doi: 10.2307/3872932

[57] Robertson PA, Bowser YH. Coarse Woody Debris in Mature Pinus ponderosa Stands in Colorado. Journal of the Torrey Botanical Society. 1999;126(3):255. doi: 10.2307/2997280

[58] NLCD 2021 USFS Tree Canopy Cover (CONUS). Salt Lake City (UT): U. S. Forest Service. 2021. https://www.mrlc.gov/data/nlcd-2021-usfs-tree-canopy-cover-conus

[59] Slope Degrees Layer. http://www.landfire/viewer. 2022. Accessed 2023 September.

[60] White GC, Burnham KP. Program MARK: survival estimation from populations of marked animals. Bird Study. 1999;46(sup1):S120–39. doi: 10.1080/00063659909477239

[61] Rota CT, Fletcher RJ, Dorazio RM, Betts MG. Occupancy estimation and the closure assumption. J Appl Ecol. 2009;46(6):1173–81.

[62] Gould MJ, Gould WR, Cain JWI, Roemer GW. Validating the performance of occupancy models for estimating habitat use and predicting the distribution of highly-mobile species: A case study using the American black bear. Biol Conserv. 2019;234:28–36.

[63] Doherty PF, White GC, Burnham KP. Comparison of model building and selection strategies. J Ornithol. 2012;152:317–23.

[64] HURVICH CM, TSAI C-L. Regression and time series model selection in small samples. Biometrika. 1989;76(2):297–307. doi: 10.1093/biomet/76.2.297

[65] Burnham KP, Anderson DR. Model selection and multimodel inference: a practical information-theoretic approach. 2nd ed. New York, NY: Springer. 2002.

[66] ARNOLD TW. Uninformative Parameters and Model Selection Using Akaike’s Information Criterion. J Wildl Manag. 2010;74(6):1175–8. doi: 10.1111/j.1937-2817.2010.tb01236.x

[67] Sutherland C, Hare D, Johnson PJ, Linden DW, Montgomery RA, Droge E. Practical advice on variable selection and reporting using Akaike information criterion. Proc Biol Sci. 2023;290(2007):20231261. doi: 10.1098/rspb.2023.1261 37752836 PMC10523071

[68] Lonsinger RC, Murley BP, McDonald DT, Fallon CE, White KM. Habitat and Predator Influences on the Spatial Ecology of Nine-Banded Armadillos. Diversity. 2025;17(4):290. doi: 10.3390/d17040290

[69] Fiske I, Chandler R. Overview of unmarked: an R package for the analysis of data from unmarked animals. https://cran.r-project.org/web/packages/unmarked/index.html. 2015.

[70] Steenweg R, Whittington J, Hebblewhite M, Forshner A, Johnston B, Petersen D. Camera-based occupancy monitoring at large scales: power to detect trends in grizzly bears across the Canadian Rockies. Biol Conserv. 2016;201:192–200.

[71] Lonsinger RC, Knight RN, Waits LP. Detection criteria and post-field sample processing influence results and cost efficiency of occupancy-based monitoring. Ecol Appl. 2021;31(7):e02404. doi: 10.1002/eap.2404 34231272

[72] Stetz JB, Seitz T, Sawaya MA. Effects of Exposure on Genotyping Success Rates of Hair Samples from Brown and American Black Bears. Journal of Fish and Wildlife Management. 2014;6(1):191–8. doi: 10.3996/122013-jfwm-085

[73] Gagneux P, Boesch C, Woodruff DS. Microsatellite scoring errors associated with noninvasive genotyping based on nuclear DNA amplified from shed hair. Mol Ecol. 1997;6(9):861–8. doi: 10.1111/j.1365-294x.1997.tb00140.x 9301074

[74] Seutin G, White BN, Boag PT. Preservation of avian blood and tissue samples for DNA analyses. Can J Zool. 1991;69(1):82–90.

[75] Frantzen MA, Silk JB, Ferguson JW, Wayne RK, Kohn MH. Empirical evaluation of preservation methods for faecal DNA. Mol Ecol. 1998;7(10):1423–8. doi: 10.1046/j.1365-294x.1998.00449.x 9787450

[76] Taberlet P, Griffin S, Goossens B, Questiau S, Manceau V, Escaravage N, et al. Reliable genotyping of samples with very low DNA quantities using PCR. Nucleic Acids Res. 1996;24(16):3189–94. doi: 10.1093/nar/24.16.3189 8774899 PMC146079

[77] Lonsinger RC, Waits LP. ConGenR: rapid determination of consensus genotypes and estimates of genotyping errors from replicated genetic samples. Conservation Genet Resour. 2015;7(4):841–3. doi: 10.1007/s12686-015-0506-7

[78] R Core Team. R: A language and environment for statistical computing. Vienna, Austria: R Foundation for Statistical Computing. 2024.

[79] Fetherston SC, Lonsinger RC, Perkins LB, Lehman CP, Adams JR, Waits LP. Genetic analysis of harvest samples reveals population structure in a highly mobile generalist carnivore. Ecol Evol. 2024;14(5):e11411. doi: 10.1002/ece3.11411 38799390 PMC11116766

[80] Varas-Nelson AC. Conservation genetics of black bears in Arizona and northern Mexico. Tucson (AZ): University of Arizona. 2010.

[81] Broquet T, Petit E. Quantifying genotyping errors in noninvasive population genetics. Mol Ecol. 2004;13(11):3601–8. doi: 10.1111/j.1365-294X.2004.02352.x 15488016

[82] Waits LP, Luikart G, Taberlet P. Estimating the probability of identity among genotypes in natural populations: cautions and guidelines. Mol Ecol. 2001;10(1):249–56. doi: 10.1046/j.1365-294x.2001.01185.x 11251803

[83] Miller CR, Joyce P, Waits LP. Assessing allelic dropout and genotype reliability using maximum likelihood. Genetics. 2002;160(1):357–66. doi: 10.1093/genetics/160.1.357 11805071 PMC1461941

[84] Anderson EC, Dunham KK. The influence of family groups on inferences made with the program Structure. Mol Ecol Resour. 2008;8(6):1219–29. doi: 10.1111/j.1755-0998.2008.02355.x 21586009

[85] Rodríguez-Ramilo ST, Wang J. The effect of close relatives on unsupervised Bayesian clustering algorithms in population genetic structure analysis. Mol Ecol Resour. 2012;12(5):873–84. doi: 10.1111/j.1755-0998.2012.03156.x 22639868

[86] Queller DC, Goodnight KF. Estimating relatedness using genetic markers. Evolution. 1989;43(2):258–75. doi: 10.1111/j.1558-5646.1989.tb04226.x 28568555

[87] PEAKALL R, SMOUSE PE. genalex 6: genetic analysis in Excel. Population genetic software for teaching and research. Molecular Ecology Notes. 2005;6(1):288–95. doi: 10.1111/j.1471-8286.2005.01155.x PMC3463245 22820204

[88] Peakall R, Smouse PE. GenAlEx 6.5: genetic analysis in Excel. Population genetic software for teaching and research--an update. Bioinformatics. 2012;28(19):2537–9. doi: 10.1093/bioinformatics/bts460 22820204 PMC3463245

[89] Van Horn RC, Altmann J, Alberts SC. Can’t get there from here: inferring kinship from pairwise genetic relatedness. Anim Behav. 2008;75(3):1173–80.

[90] Goudet J. FSTAT (Version 1.2): a computer program to calculate f-statistics. J Heredity. 1995;86(6):485–6.

[91] Raymond M, Rousset F. GENEPOP (Version 1.2): Population Genetics Software for Exact Tests and Ecumenicism. Journal of Heredity. 1995;86(3):248–9. doi: 10.1093/oxfordjournals.jhered.a111573

[92] Rice WR. Analyzing tables of statistical tests. Evolution. 1989;43(1):223–5. 28568501 10.1111/j.1558-5646.1989.tb04220.x

[93] Pritchard JK, Stephens M, Donnelly P. Inference of population structure using multilocus genotype data. Genetics. 2000;155(2):945–59. doi: 10.1093/genetics/155.2.945 10835412 PMC1461096

[94] Falush D, Stephens M, Pritchard JK. Inference of population structure using multilocus genotype data: linked loci and correlated allele frequencies. Genetics. 2003;164(4):1567–87. doi: 10.1093/genetics/164.4.1567 12930761 PMC1462648

[95] Latch EK, Dharmarajan G, Glaubitz JC, Rhodes OE. Relative performance of Bayesian clustering software for inferringpopulation substructure and individual assignment at low levels of population differentiation. Conserv Genet. 2006;7(2):295–302.

[96] Hubisz MJ, Falush D, Stephens M, Pritchard JK. Inferring weak population structure with the assistance of sample group information. Mol Ecol Resour. 2009;9(5):1322–32. doi: 10.1111/j.1755-0998.2009.02591.x 21564903 PMC3518025

[97] Evanno G, Regnaut S, Goudet J. Detecting the number of clusters of individuals using the software STRUCTURE: a simulation study. Mol Ecol. 2005;14(8):2611–20. doi: 10.1111/j.1365-294X.2005.02553.x 15969739

[98] Kopelman NM, Mayzel J, Jakobsson M, Rosenberg NA, Mayrose I. Clumpak: a program for identifying clustering modes and packaging population structure inferences across K. Mol Ecol Resour. 2015;15(5):1179–91. doi: 10.1111/1755-0998.12387 25684545 PMC4534335

[99] Banks SC, Peakall R. Genetic spatial autocorrelation can readily detect sex-biased dispersal. Mol Ecol. 2012;21(9):2092–105. doi: 10.1111/j.1365-294X.2012.05485.x 22335562

[100] Peakall R, Ruibal M, Lindenmayer DB. Spatial autocorrelation analysis offers new insights into gene flow in the Australian bush rat, Rattus fuscipes. Evolution. 2003;57(5):1182–95. doi: 10.1111/j.0014-3820.2003.tb00327.x 12836834

[101] Smouse PE, Peakall R, Gonzales E. A heterogeneity test for fine-scale genetic structure. Mol Ecol. 2008;17(14):3389–400. doi: 10.1111/j.1365-294x.2008.03839.x 18677808

[102] Shelford VE. Some Concepts of Bioecology. Ecology. 1931;12(3):455–67. doi: 10.2307/1928991

[103] Hastings A, Harrison S. Metapopulation dynamics and genetics. Annu Rev Ecol Syst. 1994;25(1):167–88.

[104] Bridle JR, Vines TH. Limits to evolution at range margins: when and why does adaptation fail?. Trends Ecol Evol. 2007;22(3):140–7. doi: 10.1016/j.tree.2006.11.002 17113679

[105] Schemnitz SD. Ecology of the scaled quail in the Oklahoma Panhandle. Wildlife Monographs. 1961;(8):3–47.

[106] Pigg J. Survey of fishes in the Oklahoma Panhandle and Harper County, northwestern Oklahoma. Proceedings of the Oklahoma Academy of Science. 1987;67:45–59.

[107] Johnson HE, Breck SW, Baruch-Mordo S, Lewis DL, Lackey CW, Wilson KR, et al. Shifting perceptions of risk and reward: Dynamic selection for human development by black bears in the western United States. Biological Conservation. 2015;187:164–72. doi: 10.1016/j.biocon.2015.04.014

[108] Speakman JR, Król E. Maximal heat dissipation capacity and hyperthermia risk: neglected key factors in the ecology of endotherms. J Anim Ecol. 2010;79(4):726–46. doi: 10.1111/j.1365-2656.2010.01689.x 20443992

[109] Rogers SA, Robbins CT, Mathewson PD, Carnahan AM, van Manen FT, Haroldson MA. Thermal constraints on energy balance, behaviour and spatial distribution of grizzly bears. Functional Ecology. 2021;35(2):398–410.

[110] Sawaya MA, Ramsey AB, Ramsey PW. American black bear thermoregulation at natural and artificial water sources. Ursus. 2017;27(2):129. doi: 10.2192/ursu-d-16-00010.1

[111] Kleeberg BA. Landscape associations and population genetics of American black bear in the Oklahoma Panhandle. Stillwater, OK: Oklahoma State University. 2024.

[112] Bard SM, Cain JW. Investigation of bed and den site selection by American black bears (Ursus americanus) in a landscape impacted by forest restoration treatments and wildfires. For Ecol Manage. 2020;460:117904.

[113] Onorato DP, Hellgren EC, Mitchell FS, Skiles JR. Home range and habitat use of American black bears on a desert montane island in Texas. Ursus. 2003;14(2):120–9.

[114] McComb WC. Ecology of coarse woody debris and its role as habitat for mammals. In: Zabel CJ, Anthony RG, editors. Mammal Community Dynamics: Management and Conservation in the Coniferous Forests of Western North America. Cambridge: Cambridge University Press. 2003. p. 374–404.

[115] Goguen CB, Fritsky RS, San Julian GJ. Effects of Brush Piles on Small Mammal Abundance and Survival in Central Pennsylvania. J Fish and Wildlife Management. 2015;6(2):392–404. doi: 10.3996/022015-jfwm-012

[116] Sánchez-Montoya MM, Guerrero-Brotons M, Miñano J, Gómez R. Effects of debris piles and pools along dry riverbeds on nutrients, microbial activity, and ground-dwelling arthropods: A Namibian ephemeral river case. J Arid Environ. 2020;175:104082. doi: 10.1016/j.jaridenv.2019.104082

[117] Wohl E, Scamardo J. Patterns of organic matter accumulation in dryland river corridors of the southwestern United States. Sci Total Environ. 2022;833:155136. doi: 10.1016/j.scitotenv.2022.155136 35405232

[118] Baruch-Mordo S, Wilson KR, Lewis DL, Broderick J, Mao JS, Breck SW. Stochasticity in natural forage production affects use of urban areas by black bears: implications to management of human-bear conflicts. PLoS One. 2014;9(1):e85122. doi: 10.1371/journal.pone.0085122 24416350 PMC3885671

[119] Parchizadeh J, Kellner KF, Hurst JE, Kramer DW, Belant JL. Factors influencing frequency and severity of human-American black bear conflicts in New York, USA. PLoS One. 2023;18(2):e0282322. doi: 10.1371/journal.pone.0282322 36827441 PMC9956656

[120] Hristienko H, McDonald JE Jr. Going into the 21Stcentury: a perspective on trends and controversies in the management of the American black bear. Ursus. 2007;18(1):72–88. doi: 10.2192/1537-6176(2007)18[72:gitsca]2.0.co;2

[121] Zack CS, Milne BT, Dunn WC. Southern oscillation index as an indicator of encounters between humans and black bears in New Mexico. Wildl Soc Bull. 2003;31(2):517–20.

[122] Garshelis DL, Baruch-Mordo S, Bryant A, Gunther KA, Jerina K. Is diversionary feeding an effective tool for reducing human–bear conflicts? Case studies from North America and Europe. Ursus. 2017;28(1):31–55. doi: 10.2192/ursu-d-16-00019.1

[123] Excoffier L, Foll M, Petit RJ. Genetic consequences of range expansions. Annu Rev Ecol Evol Syst. 2009;40(1):481–501.

[124] Costello CM. Estimates of dispersal and home-range fidelity in American black bears. J Mammal. 2010;91(1):116–21.

[125] Mokodongan D, Taninaka H, Sara L, Kikuchi T, Yuasa H, Suyama Y. Spatial autocorrelation analysis using MIG-seq data indirectly estimated the gamete and larval dispersal range of the blue coral, Heliopora coerulea, within reefs. Front Marine Sci. 2021;8:702977.

[126] Ginsberg JR, Milner-Gulland EJ. Sex-biased harvesting and population dynamics in ungulates: implications for conservation and sustainable use. Conserv Biol. 1994;8(1):157–66.

[127] Solberg EJ, Saether BE, Strand O, Loison A. Dynamics of a harvested moose population in a variable environment. J Anim Ecol. 1999;68(1):186–204.

[128] Kristensen TV, Puckett EE, Landguth EL, Belant JL, Hast JT, Carpenter C, et al. Spatial genetic structure in American black bears (*Ursus americanus*): Female philopatry is variable and related to population history. Heredity. 2018;120(4):329–41. 29234157 10.1038/s41437-017-0019-0 PMC5842220

[129] Boratyński Z. Energetic constraints on mammalian distribution areas. J Anim Ecol. 2021;90(8):1854–63. doi: 10.1111/1365-2656.13501 33884621

[130] Costello CM. The spatial ecology and mating system of black bears (Ursus americanus) in New Mexico. Bozeman (MT): Montana State University. 2008.

[131] Horner MA, Powell RA. Internal structure of home ranges of black bears and analyses of home-range overlap. J Mammal. 1990;71(3):402–10.

[132] Schenk A, Obbard ME, Kovacs KM. Genetic relatedness and home-range overlap among female black bears (Ursus americanus) in northern Ontario, Canada. Can J Zool. 1998;76(8):1511–9. doi: 10.1139/z98-075

[133] McInerny G, Travis JMJ, Dytham C. Range shifting on a fragmented landscape. Ecol Inform. 2007;2(1):1–8.

[134] Holt RD, Gomulkiewicz R. How does immigration influence local adaptation? A reexamination of a familiar paradigm. Am Nat. 1997;149(3):563–72.

[135] Alleaume-Benharira M, Pen IR, Ronce O. Geographical patterns of adaptation within a species’ range: interactions between drift and gene flow. J Evol Biol. 2006;19(1):203–15. doi: 10.1111/j.1420-9101.2005.00976.x 16405592

[136] Sexton JP, Strauss SY, Rice KJ. Gene flow increases fitness at the warm edge of a species’ range. Proceedings of the National Academy of Sciences. 2011;108(28). 10.1073/pnas.1100404108 PMC3136252 21709253

[137] Ojima DS, Conant RT, Parton WJ, Lackett JM, Even TL. Recent climate changes across the Great Plains and implications for natural resource management practices. Rangeland Ecology & Management. 2021;78:180–90.

[138] Kellermann V, van Heerwaarden B, Sgrò CM, Hoffmann AA. Fundamental evolutionary limits in ecological traits drive Drosophila species distributions. Science. 2009;325(5945):1244–6. doi: 10.1126/science.1175443 19729654

