# Supplementary material for: Landscape associations and population genetics of a generalist carnivore at a range limit
Source: PLoS One. 2025 Dec 18;20(12):e0334492. doi: 10.1371/journal.pone.0334492 (PMC12714288; doi:10.1371/journal.pone.0334492)
Supplement: S1 Fig — (PDF) [file pone.0334492.s001.pdf]

Supporting Information: S1 Figure

Landscape associations and population genetics of a generalist carnivore at a range limit

Bailey A. Kleeberg[1,#a], Robert C. Lonsinger[2], Jennifer R. Adams[3], Lisette P. Waits[3], W. Sue Fairbanks[1]

[1]Department of Natural Resource Ecology Management, Oklahoma State University, Stillwater, Oklahoma, United States of America
[2]U.S. Geological Survey, Oklahoma Cooperative Fish and Wildlife Research Unit, Oklahoma State University, Stillwater, Oklahoma, United States of America
[3]Department of Fish and Wildlife Sciences, University of Idaho, Moscow, Idaho, United States of America
[#a]Current Address: Caesar Kleberg Wildlife Research Institute, Texas A&M University - Kingsville, Kingsville, Texas, United States of America

Any use of trade, firm, or product names is for descriptive purposes only and does not imply endorsement by the U.S. Government.

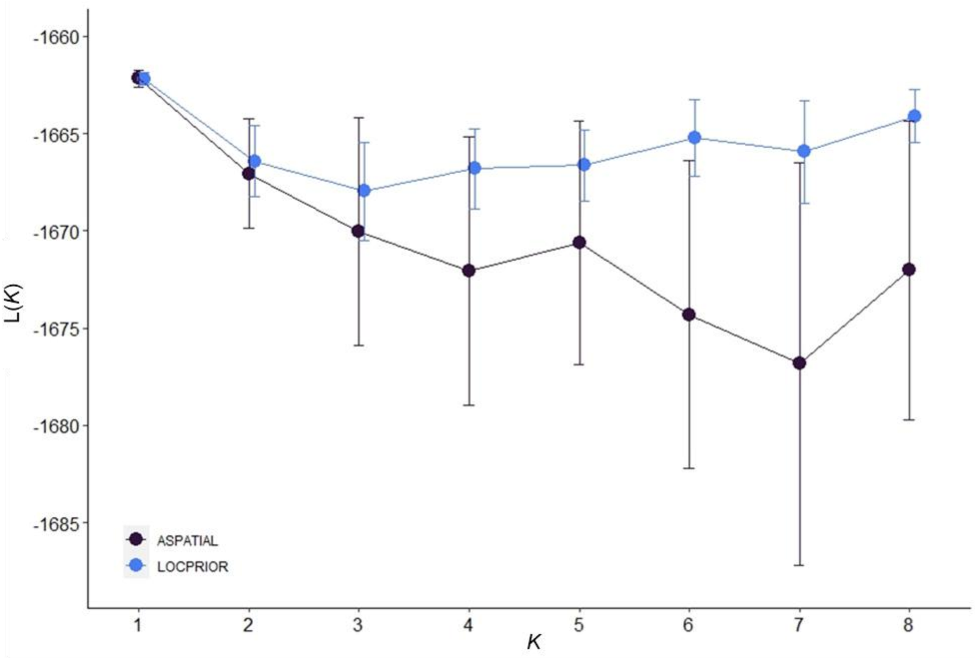

S1 Figure: The mean maximum probability value, L(*K*), with ±1 standard deviation error bars for the number of genetically distinct clusters (*K*) of black bears (*Ursus americanus*) sampled in northern New Mexico and western Oklahoma (2022–2023), based on Bayesian clustering algorithms implemented in the program STRUCTURE [1] that did (LOCPRIOR) or did not (Aspatial) consider locational data of bears as a prior.

References

1. Pritchard JK, Stephens M, Donnelly P. Inference of population structure using multilocus genotype data. Genetics. 2000;155(2):945–59.
