# Supplementary material for: Landscape associations and population genetics of a generalist carnivore at a range limit
Source: PLoS One. 2025 Dec 18;20(12):e0334492. doi: 10.1371/journal.pone.0334492 (PMC12714288; doi:10.1371/journal.pone.0334492)
Supplement: S1 Text — (PDF) [file pone.0334492.s002.pdf]

Supporting Information: S1 Text

Landscape associations and population genetics of a generalist carnivore at a range limit

Bailey A. Kleeberg[1,#], Robert C. Lonsinger[2], Jennifer R. Adams[3], Lisette P. Waits[3], W. Sue Fairbanks[1]

[1]Department of Natural Resource Ecology Management, Oklahoma State University, Stillwater, Oklahoma, United States of America
[2]U.S. Geological Survey, Oklahoma Cooperative Fish and Wildlife Research Unit, Oklahoma State University, Stillwater, Oklahoma, United States of America
[3]Department of Fish and Wildlife Sciences, University of Idaho, Moscow, Idaho, United States of America
[#a]Current Address: Caesar Kleberg Wildlife Research Institute, Texas A&M University - Kingsville, Kingsville, Texas, United States of America

Any use of trade, firm, or product names is for descriptive purposes only and does not imply endorsement by the U.S. Government.

S1 Text: Details for remote camera settings and scent lure selection for sampling black bears (*Ursus americanus*) in western Oklahoma, 2022–2023.

Remote camera settings—We set one motion-triggered Bushnell CORE™ DS-4K camera (Bushnell Corporation, Overland Park, KS) within each sampled site. We set each camera on a tree or t-post ~45 cm high (i.e., knee high) [1], parallel to the ground, and in a northerly direction. We set cameras to operate 24 hours per day, take a burst of 3 images with each trigger, and have a 10-second delay between triggers. At each site, we placed a scent pile ~5 m in front of each camera, which included medium to large rocks, sticks, and/or logs assembled into pile. We used a balanced random sampling design to select one of four lures and placed a single lure within each scent pile. Using a balanced random sampling design ensured that each lure was applied randomly to 25% of the sites (40).

Scent lure selection—To help direct black bears to within the viewsheds of our cameras, we initially identified five potential lures. We considered four lures that have previously been used—either individually or in combination with other attractants—by researchers to attract black bears: (i) a skunk-based lure (Caven's Gusto, Minnesota Trapline Products Inc., Pennock, MN) [2]; (ii) a beaver castor-based lure (Timber-Beaver Castor; Minnesota Trapline Products Inc.) [3]; (iii) an anise oil-based lure (Anise Oil, Genuine China Star, Minnesota Trapline Products Inc) [4]; and (iv) a blood-based lure [5]. We also considered (v) a blueberry-based lure (Moultrie's Bear Magnet® Liquid Attractant- Blueberry Pie, PRADCO Outdoor Brands, Birmingham, AL) used by black bear hunters. We ultimately excluded the blood-based lure due to concerns over landowner perceptions, as our Oklahoma sampling area was predominantly private lands with livestock grazing. Thus, our final lures included the (i) skunk-based lure, (ii) beaver castor-based lure, (iii) anise oil-based lure (diluted with corn oil in a 1:5 ratio), and (iv) a blueberry-based lure.

References

1. Cove MV, Kays R, Bontrager H, Bresnan C, Lasky M, Frerichs T, et al. SNAPSHOT USA 2019: A coordinated national camera trap survey of the United States. Ecology. *2021*;102(6): e03353.
2. Long RA, Donovan TM, MacKay P, Zielinski WJ, Buzas JS. Predicting carnivore occurrence with noninvasive surveys and occupancy modeling. Landscape Ecol. 2011;26(3):327–40.

3. Merkle J A., Polfus JL, Derbridge JJ, Heinemeyer KS. 2017. Dietary Niche Partitioning among black bears, grizzly bears, and wolves in a multiprey ecosystem. Can J Zool. 2017;9(9):663–671.
4. Sawaya MA., Stetz JB, Clevenger AP, Gibeau ML, Kalinowski ST. 2012. estimating grizzly and black bear population abundance and trend in Banff National Park using noninvasive genetic sampling. PLoS ONE. 2012;7(5):e34777.
5. Gould MJ, Cain III JW, Roemer GW, Gould WR, Liley SG. Density of American black bears in New Mexico. J Wildl Manage. 2018;82(4):775–88.
