# Supplementary material for: Landscape associations and population genetics of a generalist carnivore at a range limit
Source: PLoS One. 2025 Dec 18;20(12):e0334492. doi: 10.1371/journal.pone.0334492 (PMC12714288; doi:10.1371/journal.pone.0334492)
Supplement: S2 Fig — (PDF) [file pone.0334492.s003.pdf]

Supporting Information: S2 Figure

Landscape associations and population genetics of a generalist carnivore at a range limit

Bailey A. Kleeberg[1,#a], Robert C. Lonsinger[2], Jennifer R. Adams[3], Lisette P. Waits[3], W. Sue Fairbanks[1]

[1]Department of Natural Resource Ecology Management, Oklahoma State University, Stillwater, Oklahoma, United States of America
[2]U.S. Geological Survey, Oklahoma Cooperative Fish and Wildlife Research Unit, Oklahoma State University, Stillwater, Oklahoma, United States of America
[3]Department of Fish and Wildlife Sciences, University of Idaho, Moscow, Idaho, United States of America
[#a]Current Address: Caesar Kleberg Wildlife Research Institute, Texas A&M University - Kingsville, Kingsville, Texas, United States of America

Any use of trade, firm, or product names is for descriptive purposes only and does not imply endorsement by the U.S. Government.

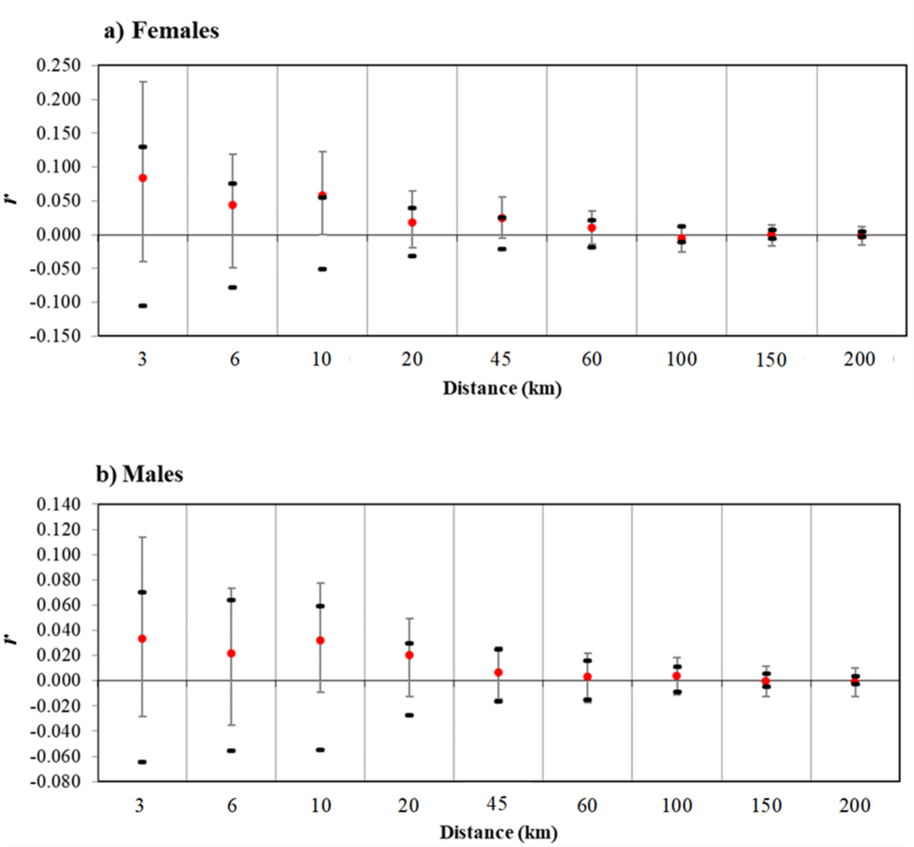

S2 Figure: Results of multiple-distance-class analyses for a) female and b) male American black bears (*Ursus americanus*) sampled in northern New Mexico and western Oklahoma (2022–2023). The estimated autocorrelation coefficient (*r*; red dot) for each distance class is presented

with 95% confidence intervals (bars) generated with bootstrap resampling; permutation tests were used to generate 95% confidence bounds (black ticks) around the null hypothesis of no spatial genetic structure. Analyses were performed with GenAlEx v6.51b2 [1,2].

References

1. Peakall R, Smouse PE. genalex 6: genetic analysis in Excel. Population genetic software for teaching and research. Mol Ecol Notes. 2006;6(1):288–95.
2. Peakall R, Smouse PE. GenAlEx 6.5: genetic analysis in Excel. Population genetic software for teaching and research—an update. Bioinformatics. 2012;28(19):2537–9.
