# Supplementary material for: Landscape associations and population genetics of a generalist carnivore at a range limit
Source: PLoS One. 2025 Dec 18;20(12):e0334492. doi: 10.1371/journal.pone.0334492 (PMC12714288; doi:10.1371/journal.pone.0334492)
Supplement: S2 Text — (PDF) [file pone.0334492.s004.pdf]

Supporting Information: S2 Text

Landscape associations and population genetics of a generalist carnivore at a range limit

Bailey A. Kleeberg[1,#a], Robert C. Lonsinger[2], Jennifer R. Adams[3], Lisette P. Waits[3], W. Sue Fairbanks[1]

[1]Department of Natural Resource Ecology Management, Oklahoma State University, Stillwater, Oklahoma, United States of America
[2]U.S. Geological Survey, Oklahoma Cooperative Fish and Wildlife Research Unit, Oklahoma State University, Stillwater, Oklahoma, United States of America
[3]Department of Fish and Wildlife Sciences, University of Idaho, Moscow, Idaho, United States of America
[#a]Current Address: Caesar Kleberg Wildlife Research Institute, Texas A&M University - Kingsville, Kingsville, Texas, United States of America

Any use of trade, firm, or product names is for descriptive purposes only and does not imply endorsement by the U.S. Government.

S2 Text: Analysis methods and results comparing support for estimates of canopy cover, slope, and elevation from six different buffer widths for estimating space use of black bears (*Ursus americanus*) in western Oklahoma, 2022–2023.

Space-use patterns of species are often scale dependent [1] and using the incorrect spatial scale for covariates can lead to biased inferences of space use patterns [2]. The scale at which black bears respond to broad-scale environmental features is not well understood in arid systems of western Oklahoma. Thus, we considered estimates of broad-scale covariates—mean canopy cover [3], slope [4], and elevation [5]—collected at six different buffer distances from 250 to 1,500 m (at 250-m intervals) around sampling sites. We used daily encounter histories of black bears detected with remote cameras to evaluate support for competing buffer distances of each remotely sensed broad-scale covariates. We selected 1,500 m as the largest buffer as it approximated the area of our occupancy sampling unit. We evaluated support for competing buffers using an occupancy modeling framework in Program MARK [6,7] by testing buffer distances for each broad-scale covariate independently. This process resulted in the creation of

six models for each broad-scale covariate and we evaluated support for each covariate independently. While evaluating support for each buffer, the occupancy (ψ) models included additive effects of the broad-scale covariate under consideration and the field-collected covariates (i.e., pricky pear [*Opuntia* spp.] cacti [PPC] count, ant mound [AM] count, coarse woody debris [CWD], and percent juniper [*Juniperus* spp.]); we held the detection (*p*) model at the global model including additive effects of lure type, temperature, precipitation, day of year (DOY), and year. We used Akaike's Information Criterion corrected for small sample size (AIC_c) to evaluate support for each model [8,9]. We retained the buffer distance for each broad-scale covariate that occurred in the most-supported model subsequent analyses.

All slope and canopy cover buffer distances were highly correlated ($r > 0.7$) and we did not include both covariates in the same model. Once we created the model set for each covariate, we tested models containing the elevation and slope buffers against models containing elevation and canopy cover to see which covariate (slope or canopy cover) was most supported by the data. Ultimately, all the slope buffer distances had more support than any canopy cover buffer distance (S2 Table 1), so we retained the most-supported slope buffer distance (1,500 m) alongside the most-supported elevation buffer distance (250 m).

S2 Table 1. Models within 5 ΔAICc of most-supported model for an analysis comparing support for estimates of canopy cover, slope, and elevation from six different buffer widths for estimating space use of black bears (*Ursus americanus*) in western Oklahoma, 2022–2023, ranked based on Akaike's information criterion with small sample size correction (AIC_c) and difference in AIC_c (ΔAIC_c), and reported with number of parameters (*K*), Akaike weight (w_i), and log-likelihood (LL).

Detection Model	Space Use Model	*K*	AIC_c	ΔAIC_c	w_i	LL
p(Global)	*ψ*(Global + E250 + S1500)	15	413.12	0.00	0.20	379.78
p(Global)	*ψ*(Global + E250 + S1250)	15	413.95	0.84	0.13	380.62
p(Global)	*ψ*(Global + E250 + S1000)	15	414.80	1.68	0.09	381.46
p(Global)	*ψ*(Global + E250 + S750)	15	415.00	1.88	0.08	381.67
p(Global)	*ψ*(Global + E250 + S250)	15	417.22	4.11	0.03	383.89
p(Global)	*ψ*(Global + E250 + S500)	15	417.24	4.13	0.03	383.91
p(Global)	*ψ*(Global + E250 + C250)	15	417.44	4.32	0.02	384.11
p(Global)	*ψ*(Global + E500 + C250)	15	417.79	4.67	0.02	384.46
p(Global)	*ψ*(Global + E750 + C250)	15	417.94	4.82	0.02	384.61
p(Global)	*ψ*(Global + E100 + C250)	15	418.00	4.88	0.02	384.67
p(Global)	*ψ*(Global + E250 + S1500)	15	413.12	0.00	0.20	379.78
p(Global)	*ψ*(Global + E250 + S1250)	15	413.95	0.84	0.13	380.62
p(Global)	*ψ*(Global + E250 + S1000)	15	414.80	1.68	0.09	381.46

Note: E= Elevation (m), S= Slope (°), C= Canopy Cover

References

1. Werdel TJ., Piper CW, Ricketts AM, Peek MS, Ahlers AA. Scale-Specific Landscape Effects Impose Range-Limiting Constraints on the Distribution of a Prairie-Obligate Carnivore. Landsc Ecol. 2022;37(8):2065–79.
2. Wiens JA. Spatial scaling in ecology. Funct Ecol. 1989;3(4):385–97.
3. NLCD 2021 USFS Tree Canopy Cover (CONUS). Salt Lake City (UT): U. S. Forest Service [USFS]. 2021 [cited 2023 Sept 9]. Available from: https://www.mrlc.gov/data/nlcd-2021-usfs-tree-canopy-cover-conus

4. Slope Degrees Layer. LANDFIRE 2.2.0. 2022 [cited 2023 Sept 9]. Available from: http://www.landfire/viewer.
5. Elevation Layer. LANDFIRE 2.2.0. 2022 [cited 2023 Sept 9]. Available from: http://www.landfire/viewer.
6. White GC, Burnham KP. Program MARK: survival estimation from populations of marked animals. Bird Study. 1999;46(sup1):S120–39.
7. MacKenzie DI, Nichols JD, Lachman GB, Droege S, Royle JA, Langtimm CA. Estimating site occupancy rates when detection probabilities are less than one. Ecology. 2002;83(8):2248–55.
8. Hurvich CM, Tsai CL. Regression and time series model selection in small samples. Biometrika. 1989;76(2):297–307.
9. Burnham KP, Anderson DR. Model selection and multimodel inference: a practical information-theoretic approach. 2nd ed. New York, NY: Springer; 2002.
