# Supplementary material for: Landscape associations and population genetics of a generalist carnivore at a range limit
Source: PLoS One. 2025 Dec 18;20(12):e0334492. doi: 10.1371/journal.pone.0334492 (PMC12714288; doi:10.1371/journal.pone.0334492)
Supplement: S3 Text — (PDF) [file pone.0334492.s005.pdf]

Supporting Information: S3 Text

Landscape associations and population genetics of a generalist carnivore at a range limit

Bailey A. Kleeberg[1,#a], Robert C. Lonsinger[2], Jennifer R. Adams[3], Lisette P. Waits[3], W. Sue Fairbanks[1]

[1]Department of Natural Resource Ecology Management, Oklahoma State University, Stillwater, Oklahoma, United States of America
[2]U.S. Geological Survey, Oklahoma Cooperative Fish and Wildlife Research Unit, Oklahoma State University, Stillwater, Oklahoma, United States of America
[3]Department of Fish and Wildlife Sciences, University of Idaho, Moscow, Idaho, United States of America
[#a]Current Address: Caesar Kleberg Wildlife Research Institute, Texas A&M University - Kingsville, Kingsville, Texas, United States of America

Any use of trade, firm, or product names is for descriptive purposes only and does not imply endorsement by the U.S. Government.

S3 Text: DNA extraction and PCR conditions for nuclear DNA amplification of black bear (*Ursus americanus*) samples collected in Oklahoma and New Mexico, 2022–2023.

We extracted DNA from hair samples using the hair protocol in the Zymo Quick-DNA Miniprep Plus Kit (Zymo Research, Irvine, CA USA) without the addition of dithiothreitol (DTT) in the proteinase K. We eluted DNA in 100 µL of the DNA Elution Buffer. We extracted DNA from fecal samples using the Zymo Quick-DNA Fecal/Soil Microbe Miniprep Kit. We extracted DNA from the hair and fecal samples in a laboratory dedicated to low quality DNA samples and included a negative control in each extraction. We extracted DNA from the tissue samples using the solid tissue protocol in the Zymo Quick-DNA Miniprep Plus Kit and we eluted DNA in 100 µL of the DNA Elution Buffer.

We tested 20 high-quality tissue samples from black bears harvested in New Mexico at 14 nuclear DNA microsatellite loci to determine the number of loci required to achieve a probability of identity for siblings (PID_{sibs}) < 0.01 in GenAlEx v6.51b2 [1,2,3]. We initially

genotyped high-quality tissue samples using a polymerase chain reaction (PCR) multiplex that contained seven nuclear DNA microsatellite loci: G10C [4], G10H [5], G10M [6,7], G10P [4], G10X [4], G1D [6,7], and Mu15 [7]. Due to low genetic diversity, we tested seven additional loci: CXX20 [8], UamD1a [9], G10B [4], G10L [4], Mu50 [6], Mu59 [10], and P07 [11]. To achieve a PID_{sibs} <0.01, consensus genotypes were required at ≥7 loci. We retained 11 loci in our final multiplex: G10C, UamD1a, G10B, G1D, Mu15, G10L, G10M, G10X, Mu59, G10H, and CXX20.

We genotyped all samples using our final multiplex with 11 microsatellite loci and one sex identification marker (SE47-48) [12]. The PCR contained 0.03 μM of G10C, 0.03 μM of UamD1a, 0.06 μM of G10B, 0.06 μM of G1D, 0.07 μM of Mu15, 0.10 μM of G10L, 0.10 μM of G10M, 0.14 μM of G10X, 0.14 μM of Mu59, 0.17 μM of G10H, 0.40 μM of CXX20, and 0.04 μM of SEY, 1× Qiagen Multiplex Kit Master Mix, 0.5 × Q solution and 2.0 mL of extracted DNA in a 7 mL reaction volume. The PCR thermal profile used for all samples included: an initial denaturation of 94°C for 15 minutes; 13 touchdown cycles at 94°C for 30 seconds (denaturation), 63°C for 90 seconds (annealing; decreasing by 0.5°C per cycle), and 72°C for 60 seconds (extension); 32 cycles at 94°C for 30 seconds (denaturation), 57°C for 90 seconds (annealing), and 72°C for 60 seconds (extension); final elongation at 60°C for 30 minutes; and cooldown at 4°C for 10 minutes. We visualized PCR products on a 3130xl Genetic Analyzer (Applied Biosystems, Foster City, CA, USA) and scored allele sizes using Genemapper 5.0 (Applied Biosystems).

References

1. Peakall R, Smouse PE. genalex 6: genetic analysis in Excel. Population genetic software for teaching and research. Mol Ecol Notes. 2006;6:288–295.
2. Peakall R, Smouse PE. GenAlEx 6.5: genetic analysis in Excel. Population genetic software for teaching and research—an update. Bioinformatics. 2012;28:2537–2539.
3. Waits LP, Luikart G, Taberlet P. Estimating the probability of identity among genotypes in natural populations: cautions and guidelines. Mol Ecol. 2001;10:249–256.
4. Paetkau, D, Calvert W, Stirling I, Strobeck C. Microsatellite analysis of population structure in Canadian polar bears. Mol Ecol. 1995;4:347–354.
5. Paetkau, D, Shields GF, Strobeck C. Gene flow between insular, coastal and interior populations of brown bears in Alaska. Mol Ecol. 1998;7:1283–1292.
6. Taberlet P, Camarra JJ, Griffin S, Uhrès E, Hanotte O, Waits LP, et al. Noninvasive genetic tracking of the endangered Pyrenean brown bear population. Mol Ecol. 1997;6:869–876.
7. De Barba M, Waits LP. Multiplex pre-amplification for noninvasive genetic sampling: Is the extra effort worth it? Mol Ecol Res. 2010;10:659–665.
8. Ostrander EA, Sprague GF, Rine J. Identification and characterization of dinucleotide repeat (CA)n markers for genetic mapping in dog. Genomics. 1993;16:207–213.
9. Meredith EP, Rodzen JA, Banks JD, Jones KC. Characterization of 29 tetranucleotide microsatellite loci in black bear (*Ursus americanus*) for use in forensic and population applications. Conserv Genet. 2009;10:693–696.
10. Bellemain E, Taberlet P. Improved noninvasive genotyping method: Application to brown bear (*Ursus arctos*) faeces. Mol Ecol Notes. 2004;4:519–522.

11. Breen M, Jouquand S, Renier C, Mellersh CS, Hitte C, Holmes NG, et al. Chromosome-specific single-locus FISH probes allow anchorage of an 1800-marker integrated radiation-hybrid/linkage map of the domestic dog genome to all chromosomes. Genome Research 2001;11:1784–1795.

12. Ennis S, Gallagher TF. A PCR-based sex-determination assay in cattle based on the bovine amelogenin locus. Anim Genet. 1994;25:425–427.
